# Support Effect of Metal–Organic Frameworks on Ethanol Production through Acetic Acid Hydrogenation

**DOI:** 10.1021/acsami.1c01100

**Published:** 2021-04-20

**Authors:** Shotaro Yoshimaru, Masaaki Sadakiyo, Nobutaka Maeda, Miho Yamauchi, Kenichi Kato, Jenny Pirillo, Yuh Hijikata

**Affiliations:** † Department of Chemistry, Faculty of Science, Kyushu University, 744 Moto-oka, Nishi-ku, Fukuoka 819-0395, Japan; ‡ Department of Applied Chemistry, Faculty of Science Division I, Tokyo University of Science, 1-3 Kagurazaka, Shinjuku-ku, Tokyo 162-8601, Japan; § International Institute for Carbon-Neutral Energy Research (WPI-I2CNER), Kyushu University, 744 Moto-oka, Nishi-ku, Fukuoka 819-0395, Japan; ∥ RIKEN SPring-8 Center,1-1-1 Kouto, Sayo-cho, Sayo-gun, Hyogo 679-5148, Japan; ⊥ Institute for Chemical Reaction Design and Discovery (WPI-ICReDD), Hokkaido University, Kita 21 Nishi 10, Kita-ku, Sapporo, Hokkaido 001-0021, Japan

**Keywords:** metal−organic framework, nanoparticle, catalysis, support effect, hydrogenation, ethanol production

## Abstract

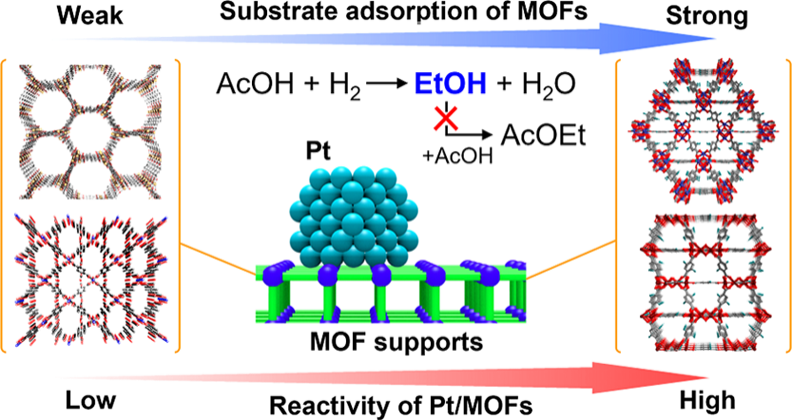

We present a systematic study on the support effect of metal–organic frameworks (MOFs), regarding substrate adsorption. A remarkable enhancement of both catalytic activity and selectivity for the ethanol (EtOH) production reaction through acetic acid (AcOH) hydrogenation (AH) was observed on Pt nanoparticles supported on MOFs. The systematic study on catalysis using homogeneously loaded Pt catalysts, in direct contact with seven different MOF supports (MIL-125-NH_2_, UiO-66-NH_2_, HKUST-1, MIL-101, Zn-MOF-74, Mg-MOF-74, and MIL-121) (abbreviated as **Pt/MOFs**), found that MOFs having a high affinity for the AcOH substrate (UiO-66-NH_2_ and MIL-125-NH_2_) showed high catalytic activity for AH. This is the first demonstration indicating that the adsorption ability of MOFs directly accelerates catalytic performance using the direct contact between the metal and the MOF. In addition, **Pt/MIL-125-NH**_**2**_ showed a remarkably high EtOH yield (31% at 200 °C) in a fixed-bed flow reactor, which was higher by a factor of more than 8 over that observed for Pt/TiO_2_, which was the best Pt-based catalyst for this reaction. Infrared spectroscopy and a theoretical study suggested that the MIL-125-NH_2_ support plays an important role in high EtOH selectivity by suppressing the formation of the byproduct, ethyl acetate (AcOEt), due to its relatively weak adsorption behavior for EtOH rather than AcOH.

## Introduction

Heterogeneous catalysis with metal catalysts loaded on support materials has greatly contributed to our industry for various chemical conversions such as the Haber–Bosch process^1,2^ and H_2_ production from methane.^3^ The interactions between the loaded metal nanoparticles (NP) and the support material have attracted much interest because they often play important roles in both catalytic activity and product selectivity in various catalyses, an effect traditionally called “support effect”.^4^ Three types of support effects have been reported to date. The first is the molecular-sieving effect that eliminates nontarget molecules from the active site on the metal NPs.^5^ The second is the charge transfer interaction, which allows support materials to change the electronic state of the metallic NPs by electron donation or withdrawal, resulting in catalytic property modulation.^6^ The third is substrate adsorption by the support materials, which would produce new reaction sites around the interface between the metal NPs and the support, to enhance catalytic activity or product selectivity.^7^ In recent years, to replace traditional oxide-based catalysts, investigations started on nontraditional solids, strongly showing such support effects. For example, Kitano et al. used an electride as the support for NH_3_ synthesis on Ru NPs to replace the traditional oxide support, MgO, through a strong electronic interaction with loaded NPs.^8^

Metal–organic frameworks (MOFs) are a new class of solids that have attracted much attention because of their designable architectures and useful applications such as gas storage,^9,10^ separation,^11,12^ drug delivery,^13,14^ and conductive materials.^15^ Many researchers have tried to apply MOFs as new catalytic supports because they possess high structural variety and are tunable due to the presence of organic components in the solid; they also have much higher thermal stability (<500 °C) than pure organic solids.^16^ The support effect of MOFs, in particular the molecular-sieving effect, has been widely reported. For example, Wang et al. reported that Pt NPs incorporated in ZIF-8 react selectively with *n*-hexene rather than with *cis*-cyclooctene.^17^ Guo et al. reported size selectivity in the hydrogenation of the reactant 1,3-cyclooctadiene in Pt/UiO-66-NH_2_ catalysts.^18^ A support effect regarding the charge transfer was also reported using the direct contact between Pt NPs and the MOF.^19^ We previously reported that catalytic activity for the CO oxidation reaction on Pt NPs is modulated by an electronic interaction between Pt and the MOF support.^19^ Li et al. also reported the effects of the electronic states of MOF supports on the catalytic property of the metal/MOF catalysts.^20,21^ However, the third support effect, substrate adsorption using the MOFs, has not been clearly reported, although MOFs are well known as specific porous solids showing various selective adsorption properties.^11^

We have focused on the clarification of the catalytic support effect of MOFs regarding substrate adsorption. In this study, we focus on the gas-phase acetic acid (AcOH) hydrogenation (AH) reaction, which is reported as a catalytic reaction on Pt NPs, requiring strong substrate adsorption by the support material.^7^ The AH produces important fundamental chemicals, such as aldehydes, esters, and alcohols. Alcohol formation, in particular, is an attractive way of producing fuels from carboxylic acids such as AcOH, which is a common product contained in bio-oil.^22^ However, selective ethanol (EtOH) production from AcOH vapor with high conversion is quite challenging because of many byproducts. The support effect regarding substrate adsorption in this reaction was reported by Rachmady et al.^7^ They found that the Pt NPs loaded on TiO_2_ that is expected to show a strong affinity for carboxylic acid exhibited the best catalytic activity and EtOH selectivity, whereas other oxide supports such as SiO_2_, Al_2_O_3_, and Fe_2_O_3_ showed low activity and selectivity.

**Figure 1:**
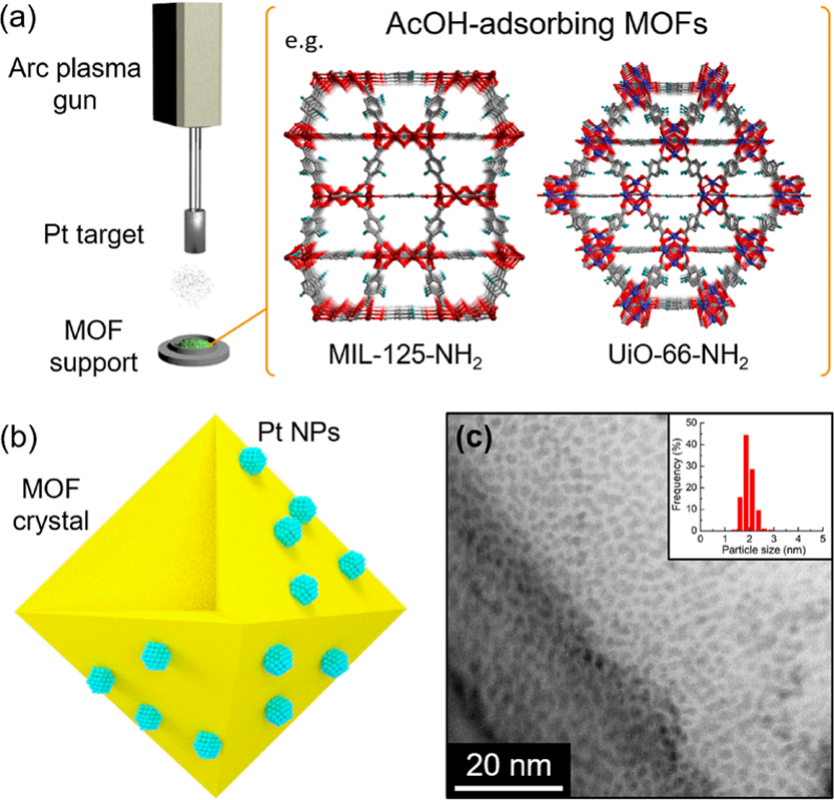
Schematic illustrations of (a) **Pt/MOF** catalyst preparation and (b) loaded Pt NPs on the MOF crystal. (c) The scanning transmission electron microscopy (STEM) image and particle distribution of **Pt/MIL-125-NH**_**2**_ (average diameter: 2.0 ± 0.2).

Here, we report the first clear demonstration of the support effect of MOFs regarding substrate adsorption, as observed in the AH reaction through direct contact between Pt and the MOF. We used Pt catalysts supported on seven different MOFs (MIL-125-NH_2_,^23^ UiO-66-NH_2_,^24^ HKUST-1,^25^ MIL-101,^26^ Zn-MOF-74,^27^ Mg-MOF-74 (CPO-27-Mg),^28^ and MIL-121^29^) (abbreviated as **Pt/MOFs**), having high tolerance against AcOH. Almost the same amount of Pt NPs having a similar diameter (approximately 0.5 wt % and 2 nm, respectively) were loaded on the MOF supports with direct contact between them, by employing the arc plasma deposition method (Figure 1a,b).^30^ We performed a systematic study on the relationship between the adsorption property of MOFs and the catalytic property of **Pt/MOFs**. By introducing an amino group (−NH_2_) as a basic site on the MOF (**Pt/MIL-125-NH**_**2**_), we observed a significant enhancement for selective EtOH production (31% yield at 200 °C), by more than a factor of 8 over that on the best oxide-based catalyst, Pt/TiO_2_,^7^ under the same conditions. This catalytic enhancement on the MOF was also investigated using in situ infrared (IR) measurements and density functional theory (DFT) calculations.

## Experimental Section

### Preparation of MOF Supports

We used **Pt/UiO-66-NH**_**2**_, **Pt/HKUST-1**, **Pt/Zn-MOF-74**, and **Pt/Mg-MOF-74** catalysts, which were previously reported in our paper for other reactions on **Pt/MOFs**.^19^ The synthetic recipe for these MOF supports and other MOFs used for a tolerance test against AcOH (Table S1) was described in the Supporting Information (SI).

### Synthesis of MIL-125-NH_2_ ({Ti_8_O_8_(OH)_4_(bdc-NH_2_)_6_}_∞_ (H_2_bdc-NH_2_ = 2-Aminoterephthalic Acid))

Titanium(IV) isopropoxide (3.12 mL, 10.4 mmol) and 2-aminoterephthalic acid (6.0 g, 36.0 mmol) were dissolved in a mixed solvent of 22.5 mL of DMF and 3.0 mL of MeOH. After sonication for 15 min, this solution was heated at 130 °C for 15 h in a Teflon-lined autoclave. The precipitate was washed with MeOH and acetone. It was filtered and dried under vacuum at 70 °C overnight.

### Synthesis of MIL-101 ({Cr_3_(OH)(H_2_O)_2_O(bdc)_3_}_∞_ (H_2_bdc = Terephthalic Acid))

Cr(NO_3_)_3_·9H_2_O (2.0 g, 5.0 mmol) and terephthalic acid (0.83 g, 5.0 mmol) were stirred in 20 mL of deionized water for 15 min. The mixture was placed in Teflon-lined autoclaves and heated at 218 °C for 18 h. The precipitate was centrifuged and washed with DMF, water, and acetone. The precipitate was dried at 70 °C under vacuum overnight.

### Synthesis of MIL-121 ({Al(OH)(H_2_btec)}_∞_ (H_4_btec = Pyromellitic Acid))

Al(NO_3_)_3_·9H_2_O (29.7 g, 79.2 mmol) and pyromellitic acid (13.4 g, 52.8 mmol) were dissolved in 80 mL of H_2_O. This solution was heated at 210 °C for 24 h in Teflon-lined autoclaves. After replacing the water, the precipitate was heated at 80 °C for 12 h. This washing process was repeated two times more. Then, the precipitate was filtered and dried in the atmosphere.

### Tolerance Test against AcOH Vapor

Each MOF support was placed in a small vial (4 mL) without a cap. The vial was put inside a larger vial (50 mL) including 5 mL of AcOH with a cap (Figure S1). The samples were exposed to AcOH vapor and kept at room temperature overnight. After that, the crystal structures of the samples were evaluated using X-ray powder diffraction (XRPD) under air.

### Preparation of Catalysts

**Pt/MIL-125-NH**_**2**_**, Pt/MIL-101**, **Pt/MIL-121**, Pt/TiO_2_ (TiO_2_: Degussa P-25), and Pt/Al_2_O_3_ (γ-Al_2_O_3_: AEROXIDE Alu C) were newly prepared in the same way as **Pt/UiO-66-NH**_**2**_, **Pt/HKUST-1**, **Pt/Zn-MOF-74**, and **Pt/Mg-MOF-74**(19) by employing the arc plasma deposition method.^30^ The loading of Pt NPs was conducted using an arc plasma gun (ULVAC ADP-3P-N2) equipping a Pt target. During the deposition, the powder of the support was put on a pot, which was continuously stirred and kept at 18 °C in a vacuum chamber. The number of plasma shots for all of the catalysts used for the AH reaction is listed in Table S2.

### Physical Measurements

XRPD patterns were measured using a Rigaku SmartLab diffractometer (Cu Kα) and synchrotron radiation (λ = 1.080 Å) at the BL44B2 RIKEN Materials Science Beamline.^31^ In the case of XRPD measurements under AcOH conditions, all **Pt/MOFs** put inside a glass capillary were preliminarily heated at 150 °C under vacuum overnight to remove water molecules in their pores. Then, AcOH vapor was introduced to the samples with saturated pressure at room temperature (*P*/*P*_0_ = 1) overnight. After exposure to AcOH vapor, the samples were sealed in the glass capillary. The samples inside the glass capillary were exposed to synchrotron radiation. Scanning transmission electron microscopy (STEM) images were collected by a JEOL JEM-ARM 200F operated at 200 kV. The loading amounts of Pt were analyzed by inductively coupled plasma-atomic emission spectroscopy (ICP-AES) using an iCAP6300 (Thermo Fisher). N_2_ adsorption isotherms were measured at 77 K using a BELSORP-max (Microtrac BEL) after complete dehydration of the samples under vacuum. X-ray photoelectron spectroscopy (XPS) was performed using a ULVAC-PHI PHI 5000 VersaProbe II (Al Kα). Temperature-programmed desorption mass spectroscopy (TPD-MS) was conducted using a BELCAT-A (Mirotrac BEL) with a quadrupole MS detector (BEL-Mass, Microtrac BEL). Before the TPD-MS measurements, each sample was put inside a glass tube and was exposed to AcOH vapor (*P*/*P*_0_ = 1) for 6 h at room temperature after complete dehydration overnight under vacuum at 150 °C. After that, the pretreated sample was transferred into a measurement tube for the BELCAT-A. TPD-MS experiments were performed at 50–300 °C (heating rate: 10 °C min^–1^) under a He flow after flowing the He gas at room temperature for stabilization of the MS detector. H_2_-pulse chemisorption measurements were conducted with the BELCAT-A (Microtrac BEL) after the pretreatment at 200 °C under a H_2_ flow.

### Catalytic Reactions

The gas-phase AH reaction was performed using 50 mg of catalysts with a homemade fixed-bed flow reactor consisting of stainless steel pipes, which were heated at 100 °C to prevent condensation of the substrate and products (Figure S2). All catalysts were pretreated at 200 °C for 2 h under a H_2_ flow. The pretreatments were carried out inside the reactor just before the catalysis to avoid exposure to air before the catalytic reaction. After the pretreatment, 30 ccm of H_2_ gas containing the saturated vapor pressure of the AcOH (10 Torr, bubbled at 17.1 °C) was introduced into the reactor (kept at 125 °C). The pressure on the reactor was then increased to 10 atm by narrowing a pressure valve located downstream of the reactor. After that, the temperature of the reactor was increased for each reaction temperature (140–280 °C). The 30 ccm of the reaction gas (3 ccm at 10 atm) was continuously introduced to the reactor from the inlet during the reaction. The online analysis of the products was performed using two gas chromatographs (a Shimadzu GC-8A with a Porapak T column on an FID detector and an active carbon column on a TCD detector) equipped downstream of the reactor. The AcOH conversion, product selectivity, and yield of ethanol (EtOH) were estimated using the following equations:
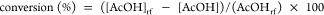

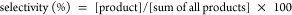

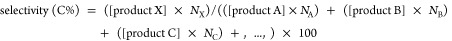

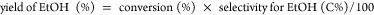
where [...] and *N*_X_ represent the concentration of the chemical species at each temperature determined by the GC and the carbon number of product X, respectively. [AcOH]_rf_ was determined by the GC under the reaction gas flow without a catalyst.

### In Situ IR measurements

In situ transmission IR spectroscopy (TIRS) was applied to monitor the transient adsorption–desorption dynamics of AcOH and EtOH on the catalytic supports. In total, 3.5 mg of the support powder was pressed into a round-shaped disc (5 mm in diameter) and placed onto a flow-through transmission cell made of quartz glass (Makuhari Rikagaku Garasu Inc.) with ZnSe windows. The ZnSe windows were heated at 60 °C with a thermostat to prevent the condensation of gaseous components such as AcOH and EtOH. Prior to IR measurements, the mounted catalyst disc was pretreated in a He flow at 300 mL min^–1^ to remove adsorbed species (mainly H_2_O). In situ TIRS spectra were recorded at 250 °C on an INVENIO R spectrometer (Bruker Optics) equipped with a liquid nitrogen-cooled mercury–cadmium–telluride (MCT) detector (D316, ZnSe Window) and an optical filter (F321). Spectra were recorded at 4 cm^–1^ of the spectral resolution and 60 kHz of the scanning velocity with 64 scans per spectrum. Modulation excitation spectroscopy (MES) was combined with in situ TIRS by periodically changing between two different gas effluents: AcOH + EtOH vapor in He balance ↔ AcOH vapor in He balance at 300 mL min^–1^. Switching between these effluents was repeated seven times. After reaching reproducible responses after two cycles, only the spectra of the last five cycles were averaged into one cycle to enhance the signal-to-noise (S/N) ratio and time resolution. The last spectrum in the period of AcOH vapor in He balance was used as a reference background.

### Theoretical Calculations

We constructed the initial structures of MIL-125-NH_2_ and UiO-66-NH_2_ using the reported structures^32,33^ where we added the missing hydrogen atoms and amino groups. All DFT calculations were performed under periodic boundary conditions and Γ-point approximation with a cutoff energy of 500 eV using the Vienna Ab Initio Simulation Package (VASP).^34,35^ A PBE functional,^36^ with projector augmented wave potentials^37^ and van der Waals interaction corrected using a D3 scheme,^38^ was used to obtain the binding energy (*E*_b,gas_) of a gas. Atomic positions were optimized by conjugate gradient methods, and convergence thresholds of the energy change and the maximum force for the optimizations were set to 10^–4^ eV and 10^–3^ eV Å^–1^, respectively. *E*_b,gas_ was defined using the following equation:
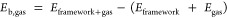
where *E*_framework+gas_ and *E*_framework_ are the energies of a framework with a gas and a framework at the optimized geometries, respectively. *E*_gas_ is the energy of the gas in an enough large supercell.

## Results and Discussion

To apply MOFs as catalytic supports for AH, we first evaluated the tolerance of MOFs against AcOH vapor because it has not been previously reported, whereas the tolerances against some solvents, acids, and bases have been reported.^16,39^ We chose 13 thermally stable MOFs, having various functional groups (e.g., −COOH, −NH_2_, and open metal sites) on the framework, as candidates for support materials, as listed in Table S1.^23−29,40−44^ In particular, we aimed at introducing basic sites, such as −NH_2_, on the framework because they have a strong affinity for acidic molecules. These MOFs were exposed to AcOH vapor in closed vials (Figure S1). Comparing XRPD patterns before and after exposure, we first found that seven MOFs, i.e., MIL-125-NH_2_, UiO-66-NH_2_, HKUST-1, MIL-101, Zn-MOF-74, Mg-MOF-74, and MIL-121, including the basic MOFs (MIL-125-NH_2_ and UiO-66-NH_2_), showed high tolerance against AcOH. We also measured N_2_ adsorption isotherms of the prepared MOFs (Figure S3). The Brunauer–Emmett–Teller (BET) surface areas of these supports were determined to be 1329 (for MIL-125-NH_2_), 1026 (UiO-66-NH_2_), 1238 (HKUST-1), 3152 (MIL-101), 1203 (Zn-MOF-74), 1113 (Mg-MOF-74), and 8.8 (MIL-121) m^2^ g^–1^, confirming their high porosity except for MIL-121, which has a bulky −COOH group inside the pore.

**Figure 2:**
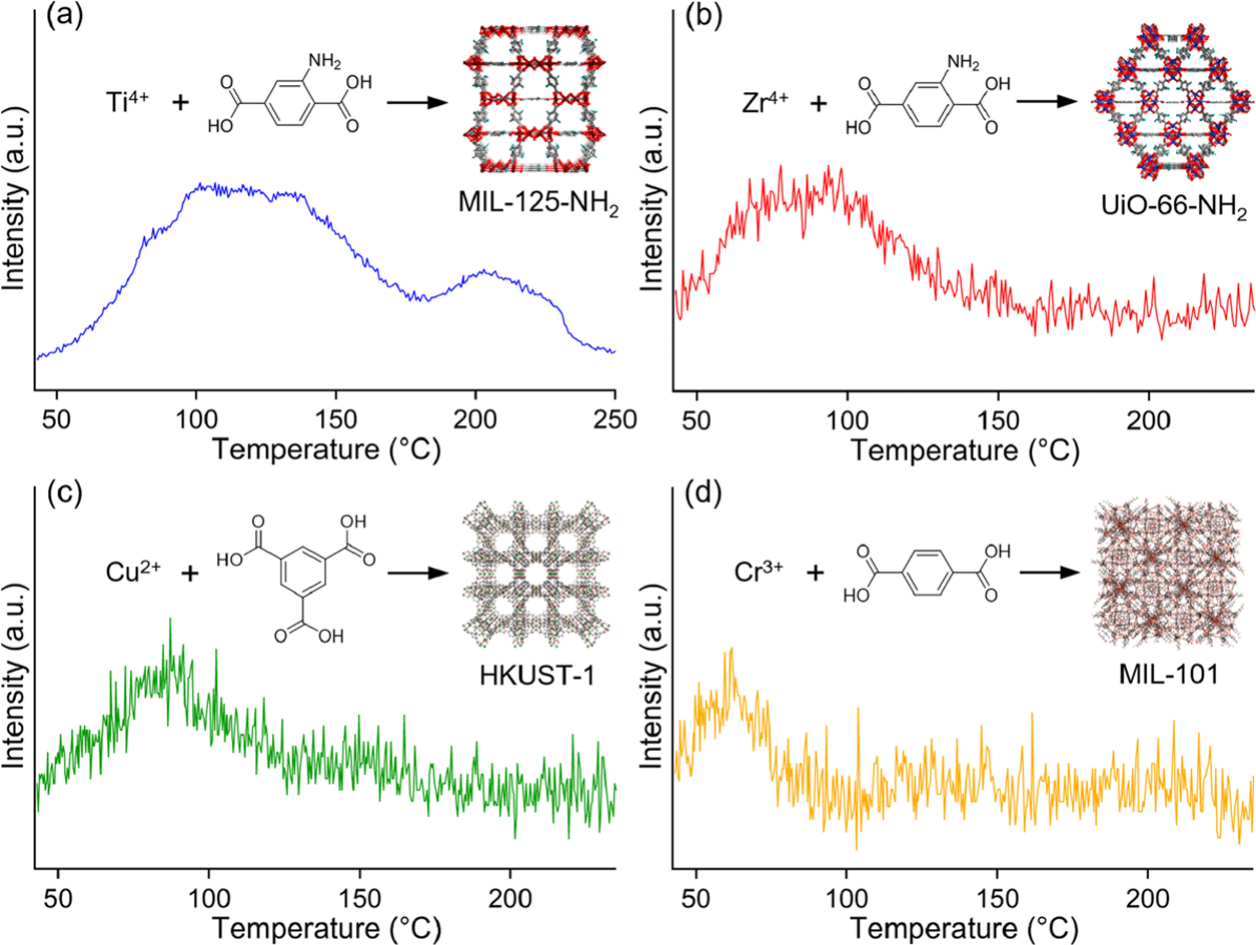
TPD-MS charts at mass number = 60 of (a) MIL-125-NH_2_, (b) UiO-66-NH_2_, (c) HKUST-1, and (d) MIL-101.

To evaluate the adsorption strength of these MOFs for the AcOH molecule, we performed TPD-MS measurements using the MOFs that were preliminarily exposed to AcOH vapor after complete dehydration. Figures 2 and S4 show the charts of TPD-MS monitoring a mass number of 60 (AcOH). Each peak observed in MIL-125-NH_2_ (<230 °C), UiO-66-NH_2_ (<160 °C), HKUST-1 (<125 °C), and MIL-101 (<80 °C) indicates desorption temperatures of the introduced AcOH under an inert gas (He) flow. The absence of a peak in Mg-MOF-74, Zn-MOF-74, and MIL-121 (Figure S4) means complete desorption under a preliminary flow of the inert gas at room temperature, showing a weak interaction with AcOH. Note that this trend was not related to the porosity of the MOFs, i.e., BET surface area, indicating that the trend relates to a difference in the interaction between the host framework and the AcOH molecule. It is clear that the desorption temperature, i.e., the adsorption strength for AcOH, strongly depends on the MOF supports. As we expected, the basic MOFs, MIL-125-NH_2_ and UiO-66-NH_2_, showed a very high affinity for AcOH, which might be derived from the acid–base or hydrogen-bonding interaction. In particular, MIL-125-NH_2_ showed the highest adsorption strength for the substrate, implying a high catalytic activity of the supported Pt NPs for AH. We also measured the temperature dependence of XRPD patterns of the MOFs under the presence of AcOH vapor at various temperatures to evaluate the stability of the MOFs for catalysis. Figure S5 shows the XRPD patterns at various temperatures under the AcOH vapor. The results showed that the framework structures of MIL-125-NH_2_, UiO-66-NH_2_, Mg-MOF-74, and MIL-121 maintained below 350 °C, while other MOFs, HKUST-1, MIL-101, and Zn-MOF-74, started to decompose above approximately 250 °C.

**Figure 3:**
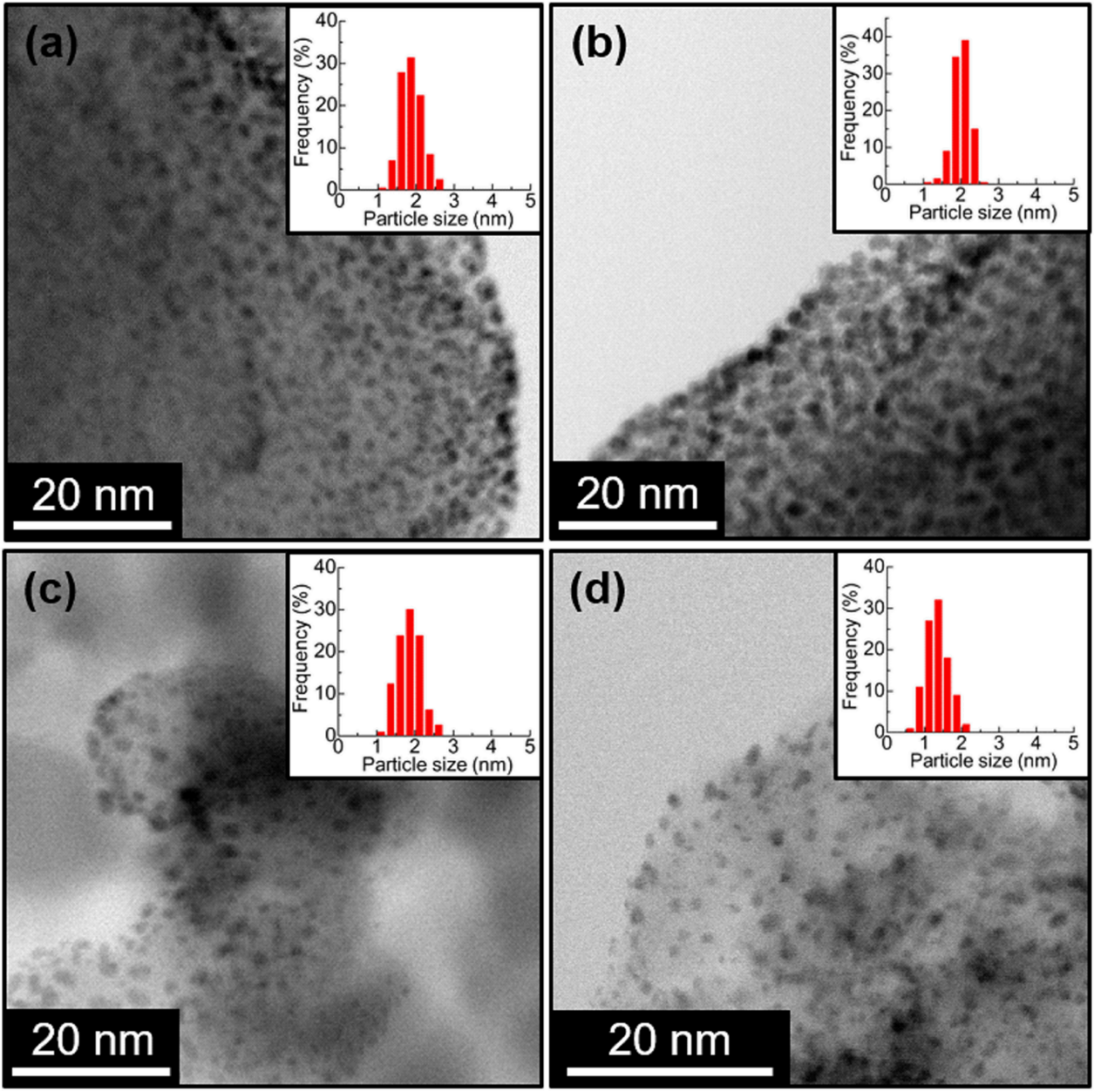
Comparison of the STEM images of **Pt/MOFs** and Pt/oxides. (a) **Pt/MIL-101** (averaged diameter: 1.9 ± 0.3 nm), (b) **Pt/MIL-121** (2.0 ± 0.2 nm), (c) Pt/TiO_2_ (1.9 ± 0.3 nm), and (d) Pt/Al_2_O_3_ (1.3 ± 0.3 nm).

As we previously reported, a systematic study on catalysis with metal NPs loaded on MOFs is not easy because various parameters, such as the NP particle size, loading amount, and protecting reagents on NPs, easily modulate the catalytic properties.^19^ To exclude these parameters, we used the arc plasma deposition method, which allowed us to deposit homogeneously distributed metal NPs having almost the same diameter (≈2 nm) without any protecting reagent, confirming the direct contact between the NPs and the MOF. We deposited Pt NPs on these MOFs with a similar loading amount. In addition to the MOF supports, we also deposited Pt NPs on TiO_2_ and Al_2_O_3_ as a traditional and control oxide support. The loading amounts were determined to be approximately 0.5 wt % for all samples using ICP-AES (Table S2). Figures 1c and 3 show STEM images of the prepared catalysts. Well-dispersed small Pt NPs having a similar diameter of around 2.0 nm (except for Pt/Al_2_O_3_ (1.3 nm)) were successfully loaded on the different supports, as is the case with **Pt/UiO-66-NH**_**2**_ (1.9 ± 0.2 nm), **Pt/HKUST-1** (2.0 ± 0.2 nm), **Pt/Zn-MOF-74** (2.0 ± 0.3 nm), and **Pt/Mg-MOF-74** (1.8 ± 0.3 nm)^19^ (Table S2). The remaining crystal structures of the support materials after the Pt loading was evaluated using XRPD (Figure S6). It is clear that the crystal structures of all samples remained even after the arc plasma deposition. Note that peaks from the loaded Pt NPs were not clearly observed because of their small diameter and low loading amount. We also measured the XRPD patterns of the prepared catalysts before and after the introduction of AcOH vapor and confirmed no change in the crystal structures (Figure S7).

**Figure 4:**
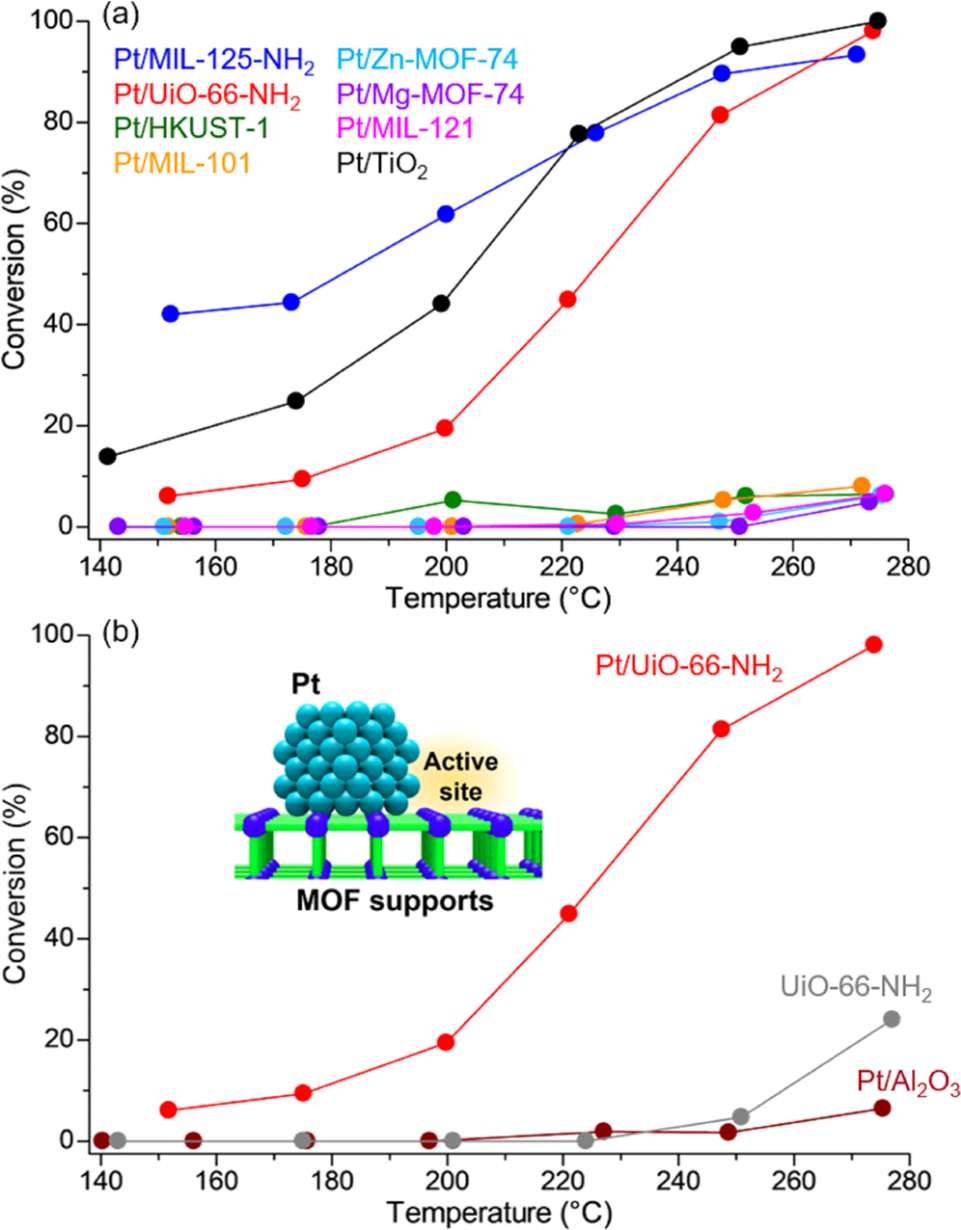
(a) AcOH conversions on **Pt/MOFs** and Pt/TiO_2_. (b) Comparison of AcOH conversions on **Pt/UiO-66-NH**_**2**_, UiO-66-NH_2_, and Pt/Al_2_O_3_.

The catalytic activity of **Pt/MOFs** and Pt/oxides for the gas-phase AH reaction was evaluated using a homemade fixed-bed flow reactor under 10 atm (Figure S2). Figure 4a shows AcOH conversions on **Pt/MOFs** and Pt/TiO_2_. The conversion at each temperature strongly depended on the support materials, confirming the presence of a strong support effect in this reaction. Pt/TiO_2_, previously reported to be the best catalyst, showed apparent conversions above 140 °C, which finally reached almost 100% at around 250 °C. In the case of **Pt/MOFs**, **Pt/MIL-125-NH**_**2**_ and **Pt/UiO-66-NH**_**2**_ including the basic MOF supports showed remarkably high conversions reaching almost 100% at around 260 °C, similar to Pt/TiO_2_. By contrast, other **Pt/MOFs** having a weak affinity for AcOH did not appear to show the catalytic activity. **Pt/MIL-125-NH**_**2**_ tends to give higher conversions in the low-temperature region compared with **Pt/UiO-66-NH**_**2**_ and other **Pt/MOFs**. The order **Pt/MIL-125-NH**_**2**_ > **Pt/UiO-66-NH**_**2**_ > other **Pt/MOFs** clearly obeys the order of the adsorption strength of the supports, observed using TPD-MS. The electronic state of the Pt^0^ site on the loaded Pt NPs was estimated using X-ray photoelectron spectroscopy (XPS) and did not show any correlation with the catalytic activity in this reaction (Figure S8 and Table S3). These results indicate that the MOF supports enhanced the catalytic activity due to their adsorption ability through the direct contact between the Pt NPs and the MOF. This is the first systematic demonstration of the third catalytic support effect of MOFs, regarding substrate adsorption, in heterogeneous catalysis. The catalytic reaction should proceed in several steps such as adsorption, activation, reaction, and desorption process.^45^ Thus, the data described above implies that the adsorption process is one of the rate-determining steps of this reaction.

Importantly, the catalytic activity on **Pt/MIL-125-NH**_**2**_ in the low-temperature region (<210 °C) is higher than that on Pt/TiO_2_, indicating that MOF-based catalysts are capable of surpassing the traditional oxide-based catalysts by the selection of optimal MOF supports. A comparison of conversions among **Pt/UiO-66-NH**_**2**_, UiO-66-NH_2_ (only support), and Pt/Al_2_O_3_ (Pt NPs on the inactive support) is shown in Figure 4b. The MOF support or loaded Pt NPs could not show a high catalytic activity by itself, suggesting that the catalytic active site on **Pt/MOFs** is located around the interface between the Pt NPs and the MOF or the area near the Pt NPs. Note that the UiO-66-NH_2_ support did not produce any EtOH even at a high temperature (270 °C) but produced acetone with high selectivity. A similar tendency was observed in **Pt/MIL-125-NH**_**2**_ (Figure S9).

**Figure 5:**
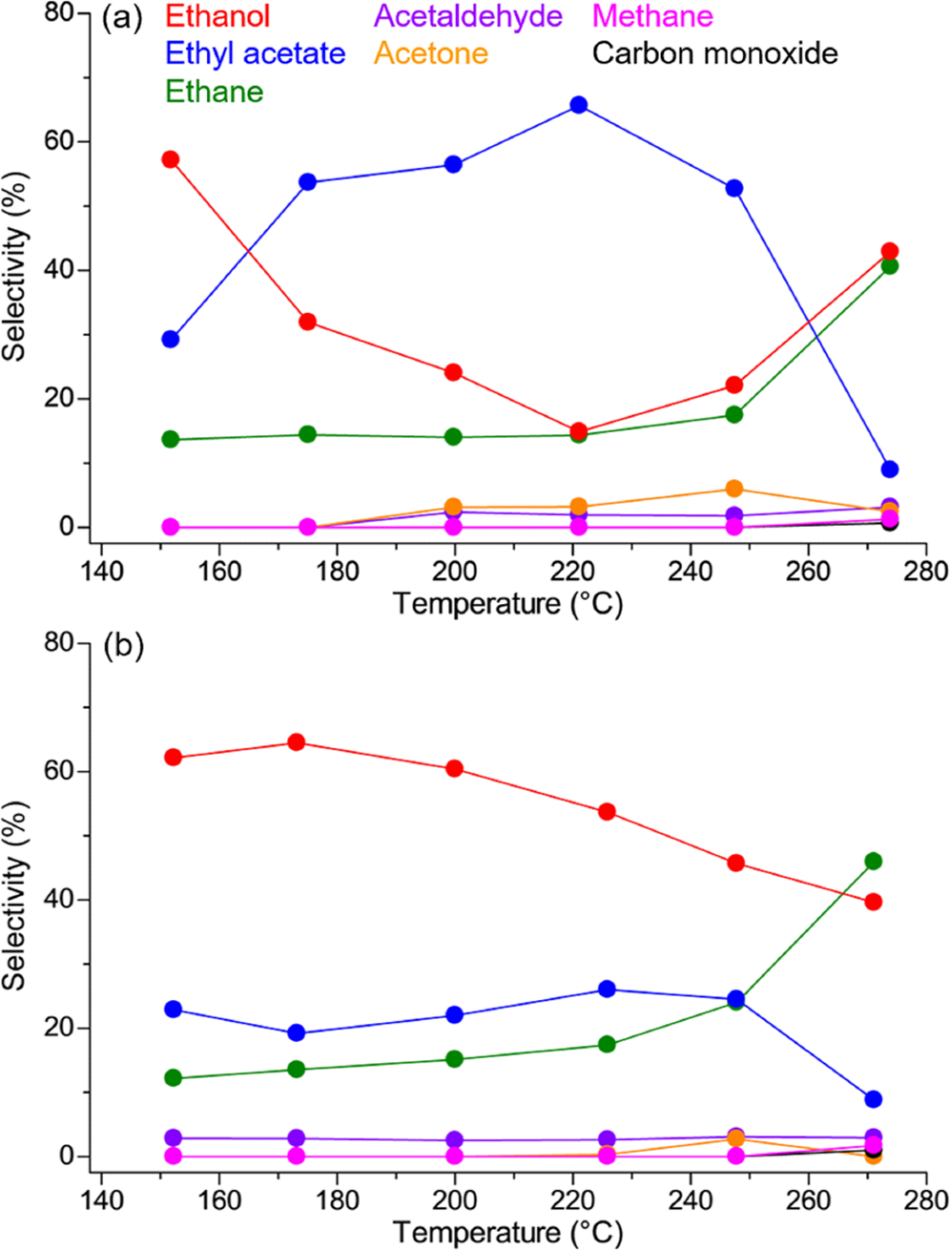
Product selectivity on (a) **Pt/UiO-66-NH**_**2**_ and (b) **Pt/MIL-125-NH**_**2**_. Red, blue, green, purple, orange, pink, and black colors correspond to ethanol, ethyl acetate, ethane, acetaldehyde, acetone, methane, and carbon monoxide, respectively.

As mentioned above, this reaction produces various byproducts in addition to EtOH. Figures 5 and S10 show the product selectivity on **Pt/MOFs** and Pt/TiO_2_. The product selectivity also greatly depended on the support materials. Focusing on the three active catalysts described above, **Pt/UiO-66-NH**_**2**_ (Figure 5a) and Pt/TiO_2_ (Figure S10f) showed a similar trend, which was different from **Pt/MIL-125-NH**_**2**_ (Figure 5b). **Pt/UiO-66-NH**_**2**_ and Pt/TiO_2_ gave high selectivity for EtOH (>40%) in the low-temperature region (<160 °C). However, the EtOH selectivity drastically decreased with increasing temperature (160 °C < *T* < 230 °C), while increasing the selectivity for ethyl acetate (AcOEt) as a byproduct. The volcano-like curve of the selectivity for AcOEt indicates that the produced EtOH on the catalysts further reacted with the remaining AcOH substrate to form AcOEt through an esterification reaction (Table S4).^7^ Surprisingly, in the case of **Pt/MIL-125-NH**_**2**_, this volcano-like curve of AcOEt selectivity was not observed and then EtOH was selectively produced as the main product (>40% selectivity) in almost the entire temperature region (140 °C < *T* < 260 °C). This result indicates that the MOF support, MIL-125-NH_2_, suppressed the formation of the AcOEt byproduct.

**Figure 6:**
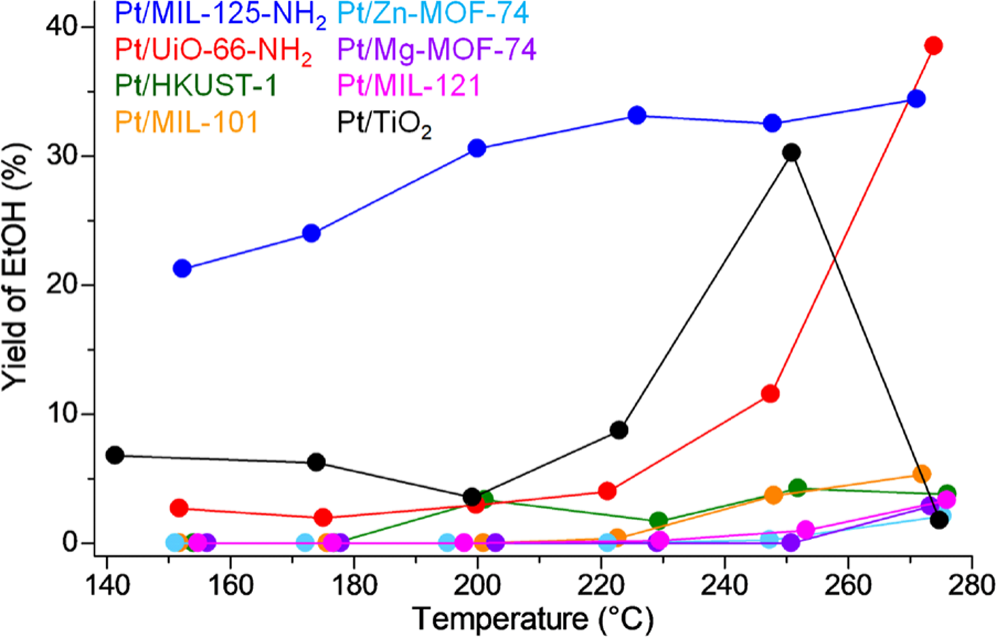
Yield of EtOH on **Pt/MOFs** and Pt/TiO_2_.

To compare the performance for EtOH production, the total yield of EtOH is plotted in Figure 6. **Pt/MIL-125-NH**_**2**_ showed the highest performance for the selective EtOH production through AH among all of the catalysts over the entire temperature region. For example, at 200 °C, **Pt/MIL-125-NH**_**2**_ showed a EtOH yield of 31%, which is more than 8 times that observed on the best oxide-based catalyst, Pt/TiO_2_ (3.5% EtOH yield). This result also indicated that MOFs have great potential in modulating the product selectivity for target molecules through direct contact with metal NPs. At the high-temperature region (>260 °C), **Pt/UiO-66-NH**_**2**_ also showed a high EtOH yield (39% at 274 °C) that is comparable to the best value on **Pt/MIL-125-NH**_**2**_ (34% at 271 °C). This high EtOH yield on **Pt/UiO-66-NH**_**2**_ should be derived from the suppression of AcOEt formation at the high temperature (Figure 5a) due to the absence of the AcOH substrate, i.e., high AcOH conversion, in this temperature region. In the case of Pt/TiO_2_, the EtOH yield increased with increasing temperature below 250 °C, which is similar to **Pt/UiO-66-NH**_**2**_ and finally reached 30% (251 °C) at a maximum. However, the EtOH yield on Pt/TiO_2_ did not further increase above 250 °C but decreased to almost 0 (1.8% at 275 °C), which is due to highly selective ethane formation on Pt/TiO_2_ at the high temperature (98% selectivity at 275 °C, Figure S10f). Table S4 shows reaction steps in AH. According to the literature,^7^ ethane production can be achieved by three major steps. The first step is that spillover hydrogens (H_sp_), generated around the adsorbed hydrogen atoms on Pt active sites, reduce the adsorbed CH_3_COOH (AcOH) to produce CH_3_CHO (acetaldehyde). The second is to produce CH_3_CH_2_OH (EtOH) through further hydrogenation of CH_3_CHO by H_sp_ around Pt. The third step is further hydrogenation of CH_3_CH_2_OH by H_sp_ around Pt to produce CH_3_CH_3_ (ethane). The difference in the product selectivity at the high-temperature region (>250 °C) between Pt/TiO_2_ and **Pt/UiO-66-NH**_**2**_ might be derived from a slight difference regarding the third step, e.g., adsorption strength for CH_3_CH_2_OH.

We also evaluated the turnover frequency (TOF) for EtOH production on each sample by determining the number of Pt sites using H_2_ chemisorption (Table S5). As shown in Figure S11, TOF on **Pt/MIL-125-NH**_**2**_ was much higher than that on Pt/TiO_2_ at almost all temperatures, confirming that this efficient EtOH production was achieved by modulating the character of each catalytic active site. **Pt/UiO-66-NH**_**2**_ showed the highest value at a high temperature (274 °C), indicating that the excellent catalytic active site is also formed through the direct contact between Pt and UiO-66-NH_2_. Although both **Pt/MIL-125-NH**_**2**_ and **Pt/UiO-66-NH**_**2**_ provided excellent catalytic active sites for EtOH production at the high temperature, there is a great difference in the catalytic performance of each site in the low-temperature region (<260 °C), i.e., active sites generated between Pt and MIL-125-NH_2_ were highly selective for EtOH production even at a low temperature.

**Figure 7:**
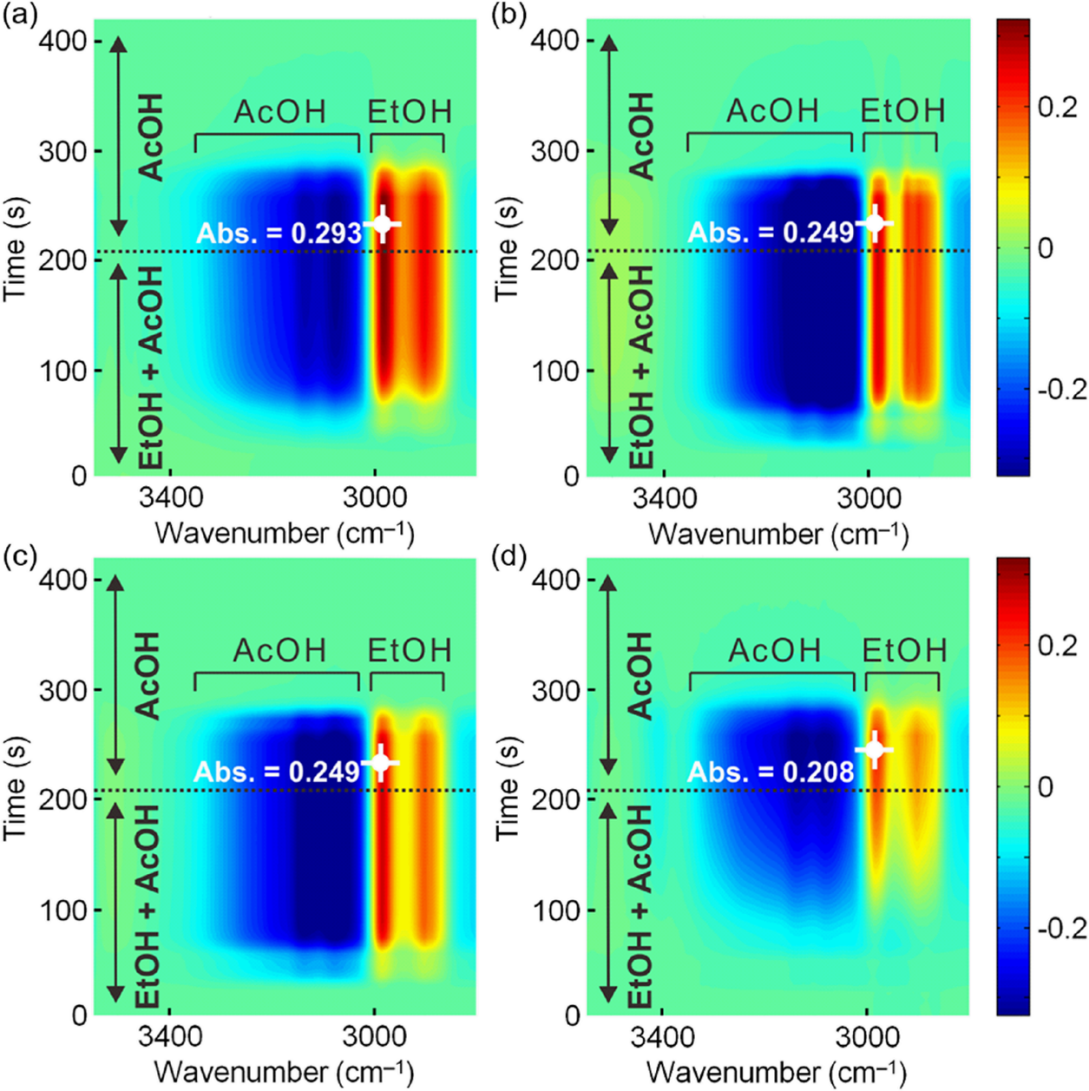
Time-domain spectra of modulation excitation IR spectroscopy for (a) Al_2_O_3_, (b) TiO_2_, (c) UiO-66-NH_2_, and (d) MIL-125-NH_2_.

To clarify underlying mechanisms for high EtOH selectivity, i.e., suppression of AcOEt formation, on **Pt/MIL-125-NH**_**2**_ at the low-temperature region (<260 °C), we performed in situ MES-IR measurements for the Al_2_O_3_, TiO_2_, UiO-66-NH_2_, and MIL-125-NH_2_ supports under the reaction gas flow at 250 °C (Figures 7 and S12). In this measurement, the time dependence of the modulation of IR spectra was monitored by repeatedly changing two different gas effluents: AcOH vapor (210–420 s) and the mixed gas of EtOH/AcOH vapors (0–210 s). The internal buffer as a background was taken in the last spectrum at 420 s when only AcOH was present. Therefore, any change observed in the spectra is such that AcOH molecules are replaced by EtOH molecules on the surface or in the pores. In Figure 7, at 0 s (i.e., after changing the AcOH flow into EtOH/AcOH (addition of the EtOH vapor)), Al_2_O_3_, TiO_2_, and UiO-66-NH_2_ exhibited an immediate decrease of the broad band at around 3200 cm^–1^ (i.e., the blue color appeared in approximately 20 s in Figure 7), which is assignable to the stretching vibration of the hydroxyl group of AcOH, with an increase in the sharp band at around 2900 cm^–1^ (the red color in Figure 7) originating from the asymmetric and symmetric stretching vibrations of the ethyl group of EtOH. This immediate decrease of the AcOH peak with an increase of the EtOH peak after the addition of EtOH vapor clearly indicates that a partial exchange of the adsorbed AcOH with EtOH immediately proceeded under the coexistence of EtOH vapor. In contrast, in the case of MIL-125-NH_2_, the changes in the AcOH (blue color) and EtOH (red color) peaks after the addition of EtOH vapor were gradually observed in approximately 100 s (Figure 7), indicating that the exchange of the adsorbed AcOH with EtOH on MIL-125-NH_2_ gradually started after 100 s, which is much slower than the others. This result suggests that MIL-125-NH_2_ tends not to adsorb EtOH strongly in the presence of AcOH. The lowest increase in absorbance at the maximum on the EtOH peak for MIL-125-NH_2_ (Abs. = 0.208) compared to other support materials (Abs. = 0.249 or 0.293) is also indicative of the weak binding for EtOH by MIL-125-NH_2_. This result implies that EtOH catalytically produced on **Pt/MIL-125-NH**_**2**_ is not strongly bound and can easily leave the catalyst, resulting in the avoidance of the further reaction with the adsorbed AcOH to form AcOEt through the esterification reaction.

To obtain more information about the difference in adsorption between MIL-125-NH_2_ and UiO-66-NH_2_, we also estimated the binding energy difference between EtOH and AcOH in these two MOFs using DFT calculations. Although the binding energies of AcOH in both MIL-125-NH_2_ and UiO-66-NH_2_ are larger than for EtOH, the energy difference in binding energy between AcOH and EtOH on MIL-125-NH_2_ was found to be 2.7 kcal mol^–1^, which is larger than that for UiO-66-NH_2_ (1.9 kcal mol^–1^). The trend of the energy difference indicates that EtOH tends to be excluded from MIL-125-NH_2_ easier than from UiO-66-NH_2_. Figure S13 shows the optimized structures of these MOFs with AcOH or EtOH. In the case of MIL-125-NH_2_, AcOH was bound by two hydrogen bonds of N–H (framework)···O (AcOH) (N···O distance is 2.920 Å) and O (framework)···H–O (AcOH) (O···O: 3.108 Å), which is different from EtOH that was bound by one hydrogen bond of O (framework)···H–O (EtOH) (O···O: 2.986 Å). In contrast, UiO-66-NH_2_ showed a similar adsorption geometry for both AcOH and EtOH molecules, containing OH−π interactions (Figure S13c,d). The larger binding energy difference of MIL-125-NH_2_ would be derived from the apparent difference in the adsorption geometry in the pore. The observed trend of binding energy differences was consistent with the results of the in situ IR measurements, suggesting that the high EtOH selectivity on **Pt/MIL-125-NH**_**2**_ is achieved by the large difference in the adsorption geometry between the AcOH substrate and the EtOH product in MIL-125-NH_2_. Although there might be other factors for varying catalytic properties on **Pt/MOFs**, such as a dangling bond on the surface or defects of MOFs, we believe that the difference in the adsorption geometry of the substrate and the product in MOFs is one of the dominant factors for the catalytic properties of **Pt/MOFs**. Considering the fact that many MOFs show unique adsorption for specific guest molecules, there should be great opportunities to find out such a large difference in adsorption strength and thus to control the catalytic properties of **Pt/MOFs**. Although we used the direct contact between the Pt NPs and the surface of the MOF crystal to perform a systematic study on the support effect in this study, we believe that it is not an ideal structure as a MOF-based composite catalyst because the inner pores of MOFs could not deeply contribute to enhancing the catalytic activity. We think that MOF-based composite catalysts with ideal structures such as Pt@MOF (Pt NPs incorporated inside the MOF) have great potential in showing much higher catalytic activity for this catalytic reaction, while oxide-based catalysts do not have such potential.

## Conclusions

In summary, we first demonstrated the third support effect of MOFs, regarding substrate adsorption, in heterogeneous catalysis. A systematic study on the EtOH production through the AH reaction using **Pt/MOFs** revealed that the catalytic active site for AH was generated through direct contact between Pt and the basic MOF and that the adsorption strength of MOFs directly modulated the catalytic activity. A relatively weak interaction between EtOH and the MIL-125-NH_2_ support suppressed the AcOEt formation in **Pt/MIL-125-NH**_**2**_, resulting in remarkably high EtOH selectivity on the **Pt/MIL-125-NH**_**2**_ catalyst, which was much better than that on the best oxide-based catalyst, Pt/TiO_2_. This remarkable enhancement of catalytic activity on metal NPs loaded on MOFs demonstrated that MOFs are one of the promising support materials as nontraditional solids. We believe that the adsorption ability of MOFs would enhance the catalytic activity and product selectivity for many other reactions and that our findings contribute to exploring new MOF-based catalysts with metal NPs.

## Supporting Information Available

Experimental details; syntheses of MOFs; preparation of **Pt/MOFs** and Pt/oxides; STEM images; XRPD patterns; XPS spectra; catalysis results; and computational details are included (PDF)

## Abbreviations Used

MOF: metal–organic framework
AH: acetic acid hydrogenation
AcOH: acetic acid
EtOH: ethanol
