# Supplementary material for: Support Effect of Metal–Organic Frameworks on Ethanol Production through Acetic Acid Hydrogenation
Source: ACS Appl Mater Interfaces. 2021 Apr 20;13(17):19992–20001. doi: 10.1021/acsami.1c01100 (PMC8288914; doi:10.1021/acsami.1c01100)
Supplement: Supplementary file 1 — am1c01100_si_001.pdf [file am1c01100_si_001.pdf]

Supporting Information

Support Effect of Metal–Organic Frameworks on Ethanol Production through Acetic Acid Hydrogenation

Shotaro Yoshimaru,[†] Masaaki Sadakiyo,[*,†,‡,§] Nobutaka Maeda,[§,¶] Miho Yamauchi,[†,§] Kenichi Kato,[#] Jenny Pirillo,[‖] Yuh Hijikata[‖]

†: Department of Chemistry, Faculty of Science, Kyushu University, 744 Moto-oka, Nishi-ku Fukuoka 819-0395, Japan

‡: Department of Applied Chemistry, Faculty of Science Division I, Tokyo University of Science, 1-3 Kagurazaka, Shinjuku-ku, Tokyo 162-8601, Japan

§: International Institute for Carbon-Neutral Energy Research (WPI-I2CNER), Kyushu University, 744 Moto-oka, Nishi-ku Fukuoka 819-0395, Japan

#: RIKEN SPring-8 Center, 1-1-1 Kouto, Sayo-cho, Sayo-gun, Hyogo 679-5148, Japan

‖: Institute for Chemical Reaction Design and Discovery (WPI-ICReDD), Hokkaido University, Kita 21 Nishi 10, Kita-ku, Sapporo, Hokkaido 001-0021, Japan

Present address: ¶ Institute of Materials and Process Engineering (IMPE), Zü-rcher Hochschule für Angewandte Wissenschaften (ZHAW), Forschungsbereich Verfahrenstechnik Technikumstrasse 9, 8400 Winterthur, Switzerland

E-mail: sadakiyo@rs.tus.ac.jp

Preparation of MOF supports

Synthesis of UiO-66-NH$_2$ ($\{Zr_6O_4(OH)_4(bdc\text{-}NH_2)_6\}_\infty$)[1]

$ZrCl_4$ (11.52 g, 49.43 mmol) was dissolved in 2880 ml DMF by sonication for 15 minute. 2-aminoterephthalic acid (8.928 g, 49.29 mmol) was added into the solution. After sonication for 15 minute, the mixture was heated at 120 °C for 24 h. The yellow precipitate was collected by centrifugation. It was washed with DMF and MeOH, and dried under vacuum at 160 °C for a night.

Synthesis of HKUST-1 ($\{Cu_3(btc)_2\}_\infty$ (H_3btc = trimesic acid))[1]

$Cu(NO_3)_2 \cdot 3H_2O$ (20.8 g, 86.0 mmol) was dissolved in a mixed solvent (500 ml, DMF : EtOH : distilled water = 1 : 1 : 1). Trimesic acid (10.0 g, 47.6 mmol) was also dissolved in the mixture and sonicated for 15 minute. The solution was heated at 85 °C for 20 h. The precipitate was separated through decantation. After washing with DMF, the blue precipitate was immersed in chloroform for four days. This operation was repeated three times. The precipitate was separated by filtration and washed with MeOH and distilled water. To dry the powder, it was heated at 160 °C in vacuo. To remove unreacted chemicals and solvent,

reflux using MeOH was conducted for a day. After that, the sample was moved into DMF. The mixture was kept at 70 °C for 26 h. After separation by centrifugation, the resultant was washed with MeOH and head at 160 °C under vacuum until dryness.

Synthesis of Zn-MOF-74 ($\{Zn_2(dobdc)\}_\infty$ (H_4dobdc = 2,5-dihydroxyterephthalic acid))[1]

$Zn(NO_3)_2 \cdot 6H_2O$ (22.6 g, 76.0 mmol) was dissolved in 1000 ml DMF. 2,5-dihydroxyterephthalic acid (5.00 g, 26.0 mmol) was also dissolved in the mixture and sonicated 10 minute. 50 ml of distilled water was added to this solition and heated at 100 °C for 20 h. The precipitate was separated by decantation and washed with DMF. It was immersed in MeOH for five days. This operation was repeated two times. The precipitate was separated by filtration. After washing with MeOH and distilled water, it was refluxed in MeOH for 30 hours. The sample was dried in air after separation by centrifugation.

Synthesis of Mg-MOF-74 (COP-27-Mg) ($\{Mg_2(dobdc)\}_\infty$)[1]

$Mg(NO_3)_2 \cdot 6H_2O$ (31.4 g, 122 mmol) was dissolved in a mixed solvent (198 ml of distilled water, 198 ml of EtOH and 2970 ml of DMF). 2,5-dihydroxyterephthalic acid (7.39 g, 37.3 mmol) was dissolved in the mixture and sonicated for 15 min. The solution was heated at 125 °C for 21 h. The precipitate was separated by decantation and immersed in MeOH for two days. This operation was repeated five times. The sample was separated by filtration and dried at 250 °C for 6 h under vacuum.

Synthesis of DUT-5 ($\{Al(OH)(bpydc)\}_\infty$ (H_2bypdc = 2,2′- bipyridyl-5,5′-dicarboxylic acid))

$Al(NO_3)_3 \cdot 9H_2O$ (1.88 g, 5.0 mmol) and H_2bpydc (0.8 g, 3.3 mmol) were dissolved in 120 ml of DMF. After sonication, the solution was placed in Teflon-lined autoclaves and heated at 180 °C for 24 h. The precipitate was collected and washed with MeOH. The white powder was dried at 120 °C under vacuum for a night.

Synthesis of UiO-66 ($\{Zr_6O_4(OH)_4(bdc)_6\}_\infty$)

$ZrCl_4$ (1.66 g, 7.1 mmol) and terephthalic acid (1.16 g, 7.0 mmol) were dissolved in 30 ml of DMF and 0.8 ml of 35% HCl. The mixture was heated and stirred at 60 °C for complete dissolution. After that, it was put in Teflon-lined autoclaves and heated at 220 °C for 17h. Resulting white precipitate was separated by filtration and washed with DMF and MeOH. The white powder was dried at 180 °C under vacuum over a night.

Synthesis of UiO-67 ($\{Zr_6O_4(OH)_4(bpdc)_6\}_\infty$ (H_2bpdc = 4,4'-biphenyldicarboxylic acid))

$ZrCl_4$ (2.3 g, 9.7 mmol) was mixed into 300 ml of DMF and stirred until completely dissolved. 4,4'-biphenyldicarboxylic acid (2.4 g, 10.0 mmol) was added into the mixture and stirred for 20 min. Then, 0.25 ml of H_2O and 50 ml of DMF were also added into the mixture. The mixture was heated at 95 °C for 99 h without stirring. After cooling to room temperature, the precipitate was filtrated and washed with ethanol. The resulting powder was dried at 90 °C under vacuum for 3h.

Synthesis of MIL-125 ($\{Ti_8O_8(OH)_4(bdc)_6\}_\infty$)

Titanium (IV) isopropoxide (0.78 ml, 2.6 mmol), terephthalic acid (1.50g, 9.0 mmol) were added in mixture of 22.5 ml of DMF and 3.0 ml of ethanol. The mixture was stirred until dissolved. Then, it was heated at 130 °C for 15 h in Teflon-lined autoclaves. The precipitate was separated by filtration and washed with MeOH and acetone. At last, the resulting white powder was heated at 70 °C under vacuum.

Synthesis of ZIF-8 ($\{Zn(mIm)_2\}_\infty$ (HmIm = 2-methylimidazole))

$Zn(NO_3)_2{\cdot}6H_2O$ (9.70 g, 33.3 mmol) and 2-methylimidazole (22.00 g, 266.6 mmol) were dissolved in 1000 ml of MeOH. The mixture was stayed for 24 h without stirring. The precipitate was separated and washed by centrifugation. The sample was dried under vacuum at room temperature.

Synthesis of ZIF-67 ($\{Co(mIm)_2\}_\infty$)

$Co(NO_3)_2{\cdot}6H_2O$ (9.70 g, 33.3 mmol) and 2-methylimidazole (22.00 g, 266.6 mmol) were dissolved in 1000 ml of MeOH. The mixture was stayed for 24 h without stirring. The precipitate was separated and washed by centrifugation. The sample was dried under vacuum at room temperature.

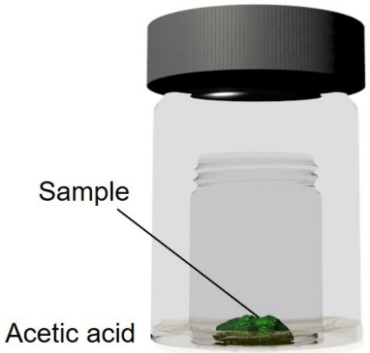

Figure S1. Schematic illustration of the tolerance test for MOFs against AcOH.

Table S1. List of the tolerances of MOFs against AcOH.

MOF	Tolerance
MIL-125	×
MIL-125-NH_2	○
UiO-66	×
UiO-66-NH_2	○
UiO-67	×
HKUST-1	○
MIL-101	○
Zn-MOF-74	○
Mg-MOF-74	○
MIL-121	○
DUT-5	×
ZIF-8	×
ZIF-67	×

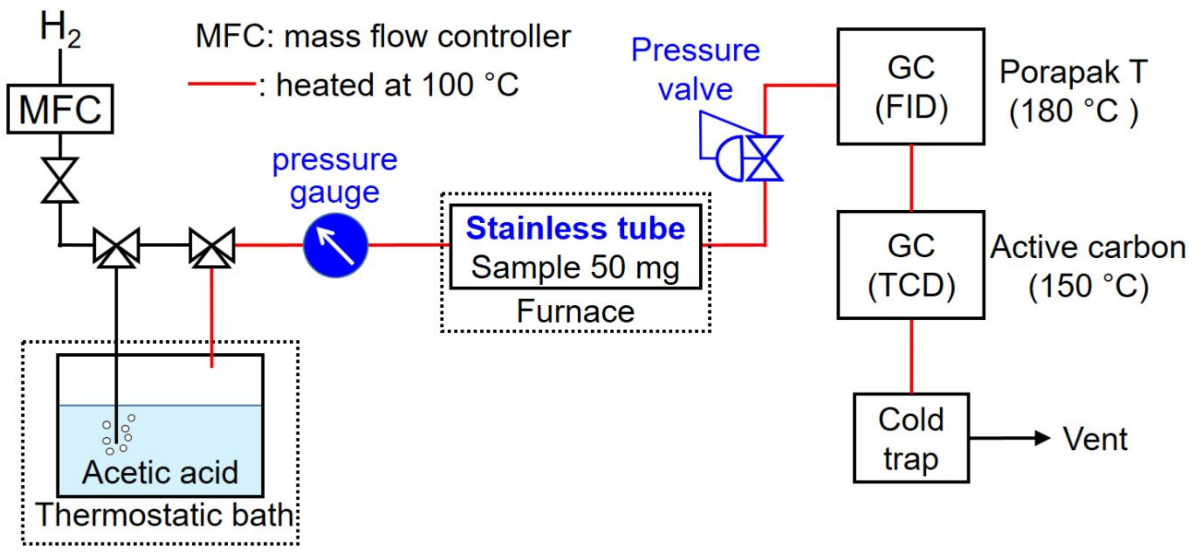

Figure S2. Home-made fixed-bed flow reactor for online gas analysis of the AH reaction

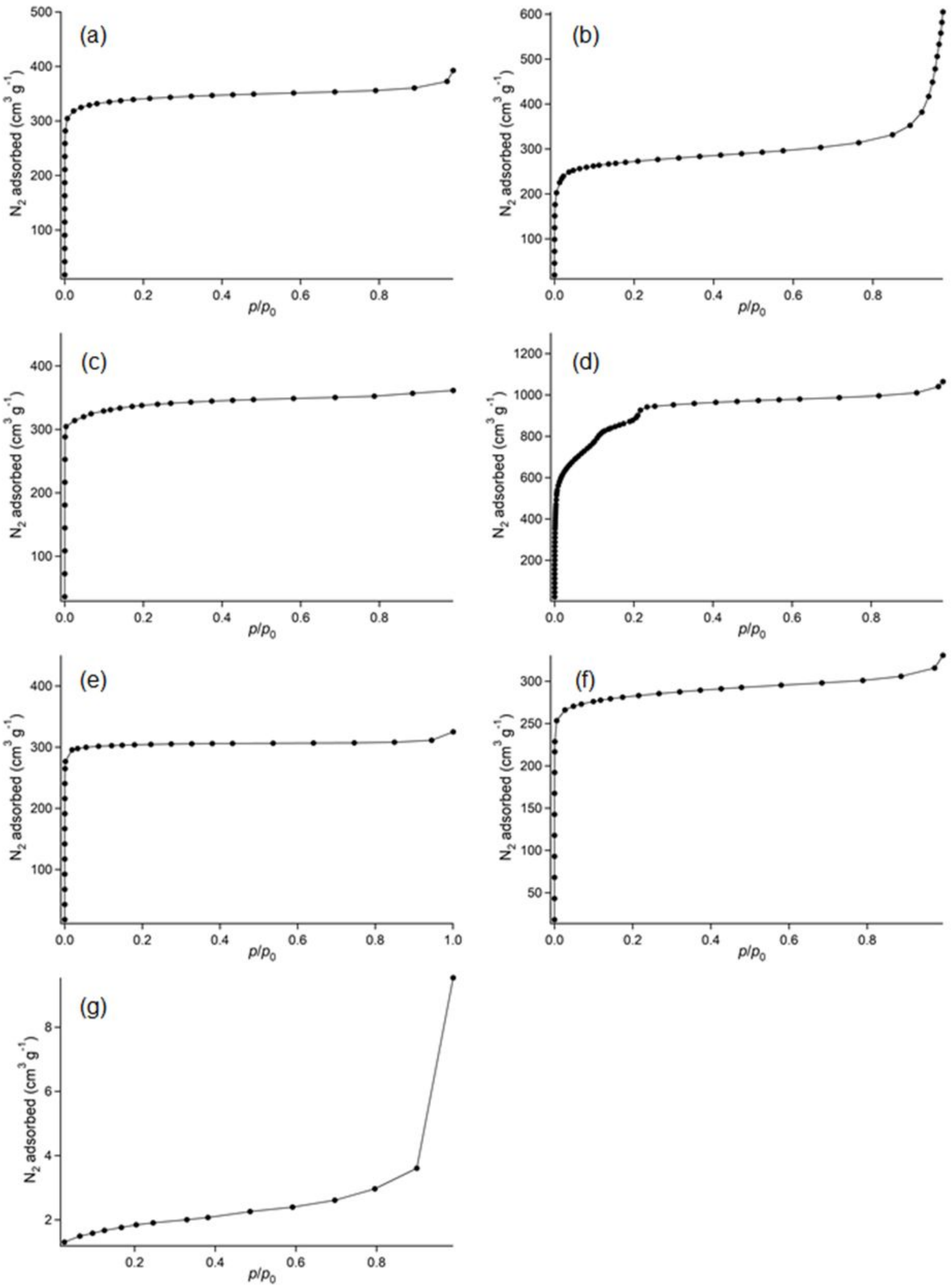

Figure S3. N_2 adsorption isotherms of (a) **MIL-125-NH$_2$**, (b) **UiO-66-NH$_2$**, (c) **HKUST-1**, (d) **MIL-101**, (e) **Zn-MOF-74**, (f) **Mg-MOF-74**, and (g) **Pt/MIL-121** at 77 K.

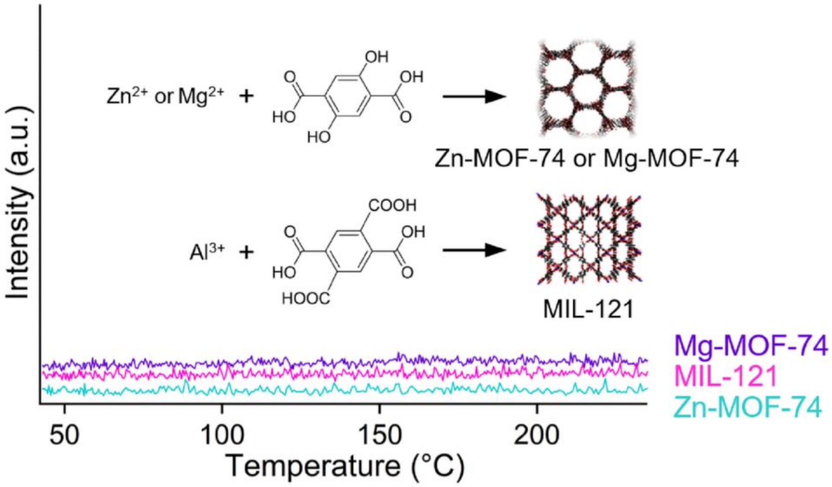

Figure S4. Charts of the TPD-MS (at mass number = 60) of Zn-MOF-74, Mg-MOF-74, and MIL-121.

Table S2. Preparation details of all the catalysts used for AH reaction. Support amount used for the arc plasma deposition, number of shots of the arc plasma gun, loading amount of Pt, and diameter of the loaded Pt NPs are listed. **Pt/UiO-66-NH$_2$**, **Pt/HKUST-1**, **Pt/Zn-MOF-74**, and **Pt/Mg-MOF-74** were already reported in our previous literature.[1]

	Pt/MIL-125-NH$_2$	Pt/UiO-66-NH$_2$	Pt/HKUST-1	Pt/MIL-101	Pt/Zn-MOF-74
Support (g)	1.83	6.05	4.45	2.78	7.32
Shot	2600	8600	5200	2800	6600
Pt (wt%)	0.56	0.44	0.47	0.58	0.48
Diameter (nm)	2.0 ± 0.2	1.9 ± 0.2	2.0 ± 0.2	1.9 ± 0.3	2.0 ± 0.3
	Pt/Mg-MOF-74	Pt/MIL-121	Pt/TiO$_2$	Pt/Al$_2$O$_3$	
Support (g)	7.28	3.46	2.05	2.15	
Shot	8200	6500	7500	8000	
Pt (wt%)	0.41	0.41	0.50	0.52	
Diameter (nm)	1.8 ± 0.3	2.0 ± 0.2	1.9 ± 0.3	1.3 ± 0.3	

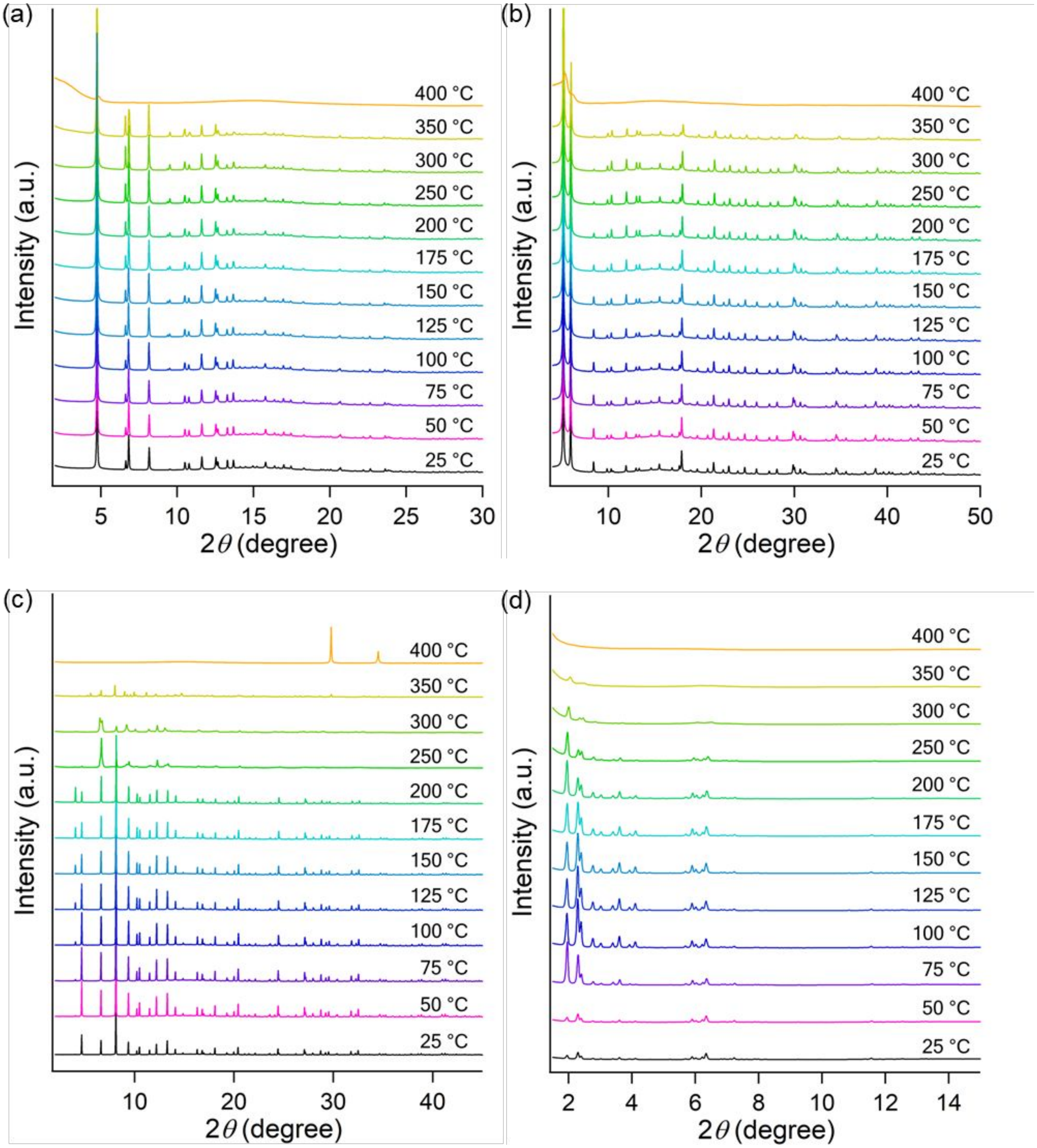
(a)
(b)
(c)
(d)
400 °C
350 °C
300 °C
250 °C
200 °C
175 °C
150 °C
125 °C
100 °C
75 °C
50 °C
25 °C
Intensity (a.u.)
2θ (degree)

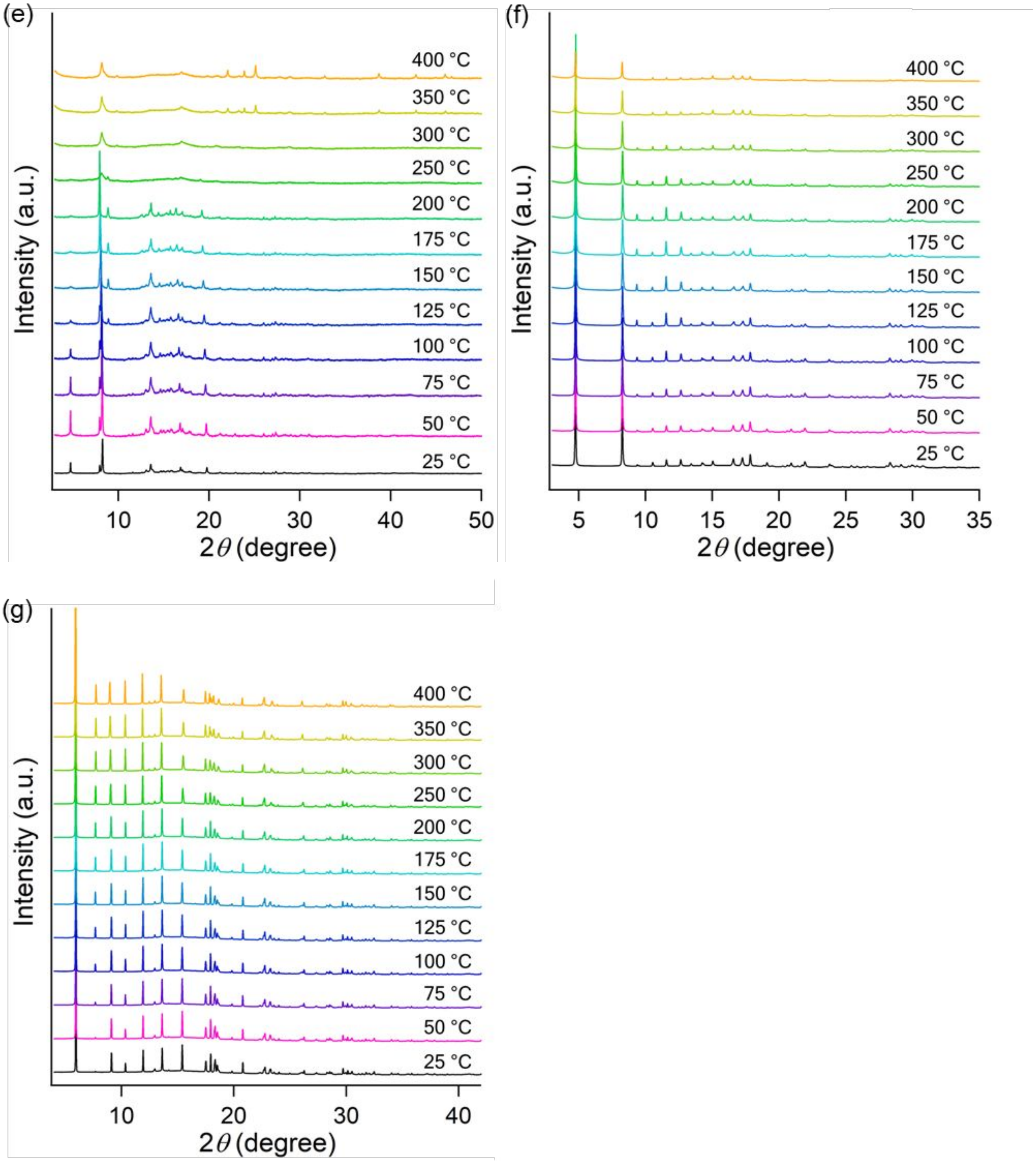

Figure S5. Temperature dependence of XRPD patterns of (a) **MIL-125-NH$_2$**, (b) **UiO-66-NH$_2$**, (c) **HKUST-1**, (d) **MIL-101**, (e) **Zn-MOF-74**, (f) **Mg-MOF-74**, and (g) **MIL-121** under AcOH vapor (λ = 1.080 Å).

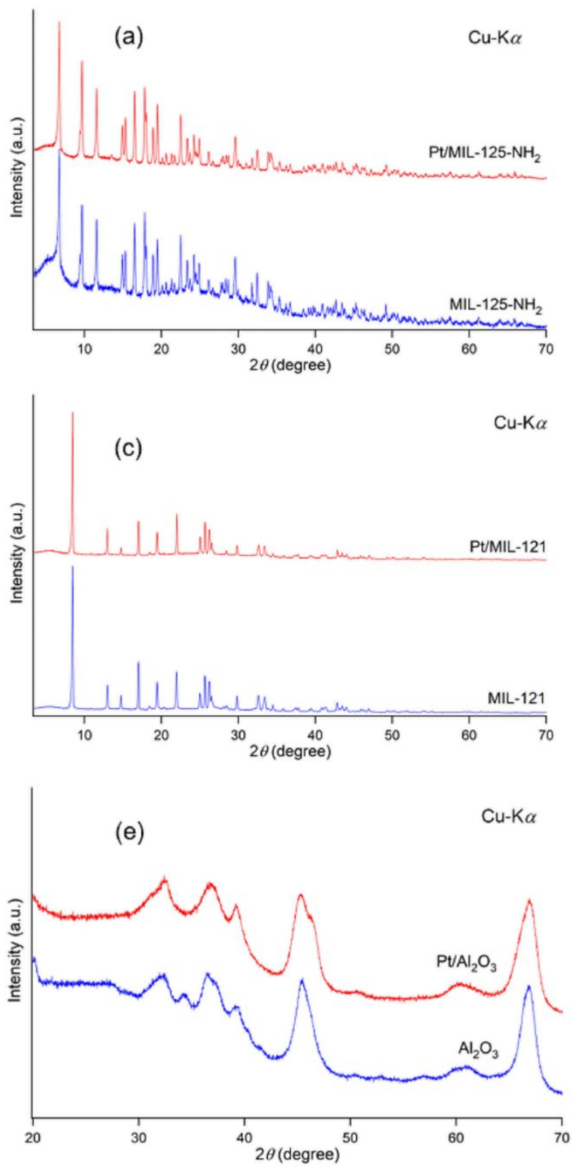

Figure S6. XRPD patterns of (a) **Pt/MIL-125-NH$_2$**, (b) **Pt/MIL-101**, (c) **Pt/MIL-121**, (d) **Pt/TiO$_2$**, and (e) **Pt/Al$_2$O$_3$** before and after the APD experiments.

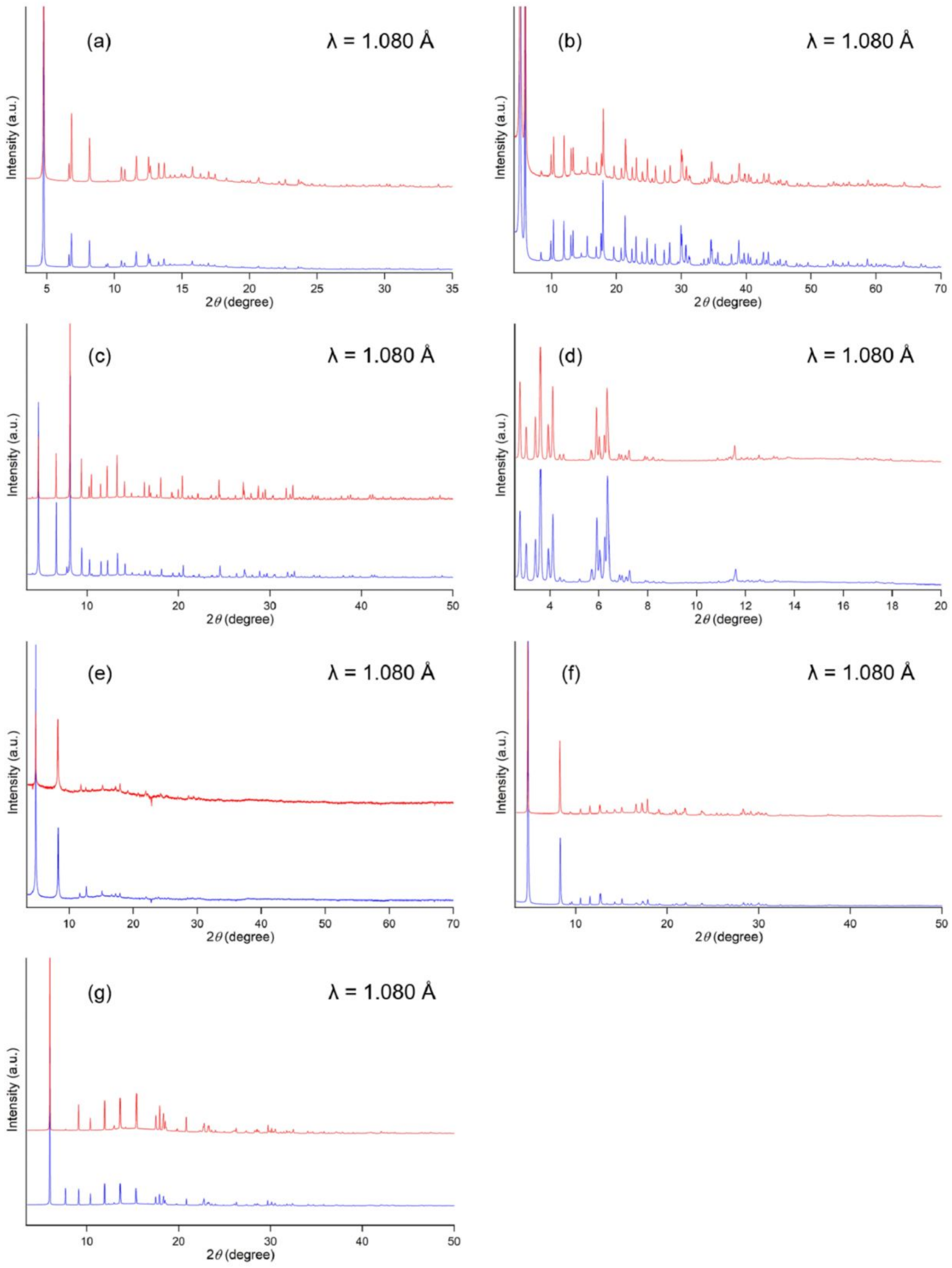

Figure S7. XRPD patterns before (blue) and after (red) AcOH introduction into (a) **Pt/MIL-125-NH$_2$**, (b) **Pt/UiO-66-NH$_2$**, (c) **Pt/HKUST-1**, (d) **Pt/MIL-101**, (e) **Pt/Zn-MOF-74**, (f) **Pt/Mg-MOF-74**, and (g) **Pt/MIL-121**.

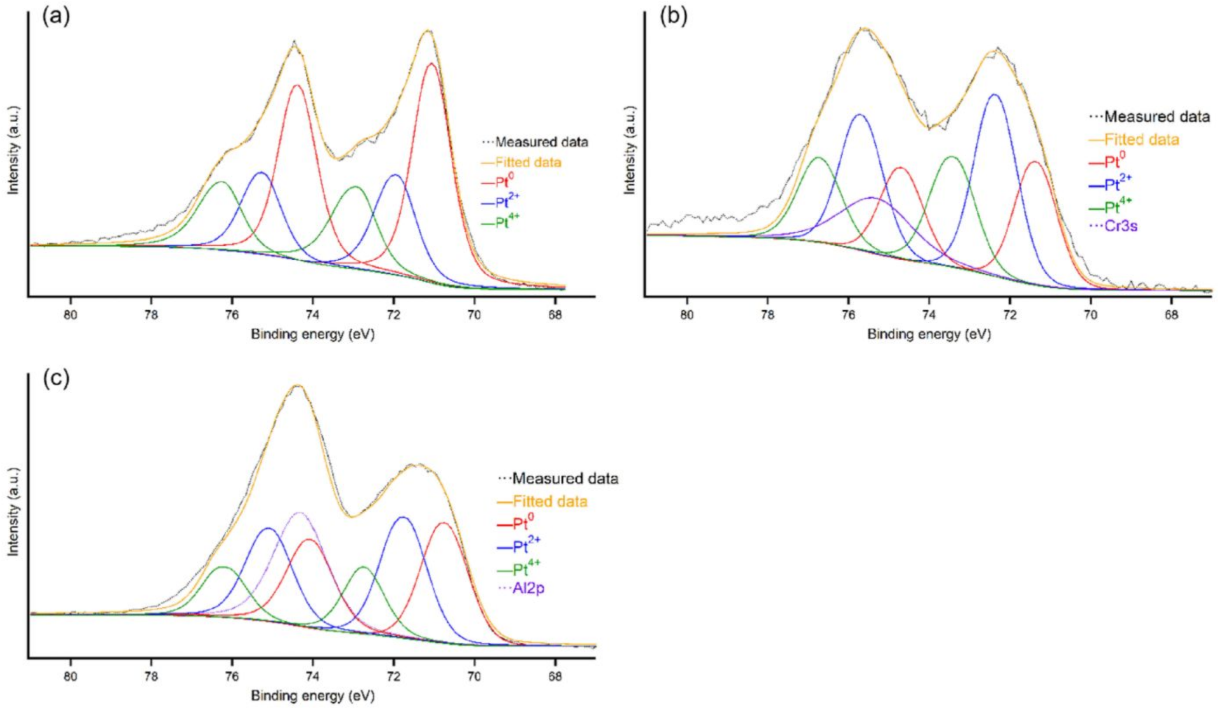

Figure S8. Comparison of XPS spectra of (a) **Pt/MIL-125-NH$_2$**, (b) **Pt/MIL-101**, and (c) **Pt/MIL-121**.

Table S3. Comparison of binding energies of Pt^0 $4f_{7/2}$ of **Pt/MOFs** determined from XPS measurements.

Pt/MOF	Binding energy (eV)
Pt/MIL-125-NH$_2$	71.03
Pt/UiO-66-NH$_2$ [1]	71.72
Pt/HKUST-1 [1]	71.21
Pt/MIL-101	71.35
Pt/Zn-MOF-74 [1]	70.83
Pt/Mg-MOF-74 [1]	71.07
Pt/MIL-121	70.74

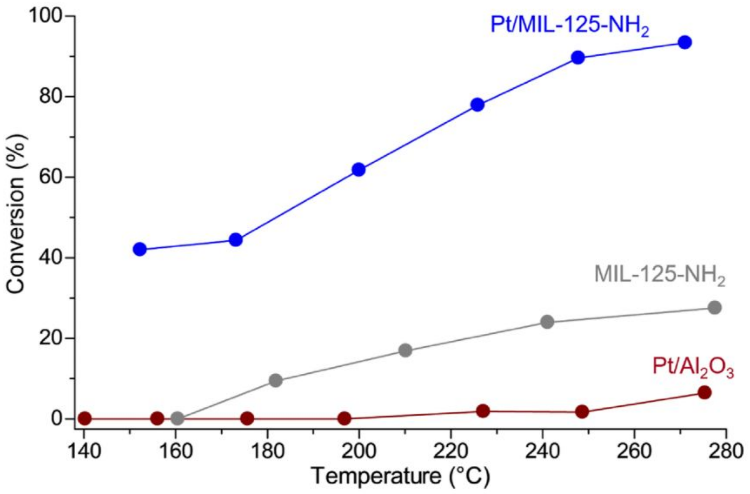

Figure S9. Comparison of AcOH conversions on **Pt/MIL-125-NH$_2$**, MIL-125-NH$_2$ (only support), and Pt/Al_2O_3 (Pt NPs on an inactive support). Note that the MIL-125-NH$_2$ support did not produce any EtOH but produced ethyl acetate and acetaldehyde.

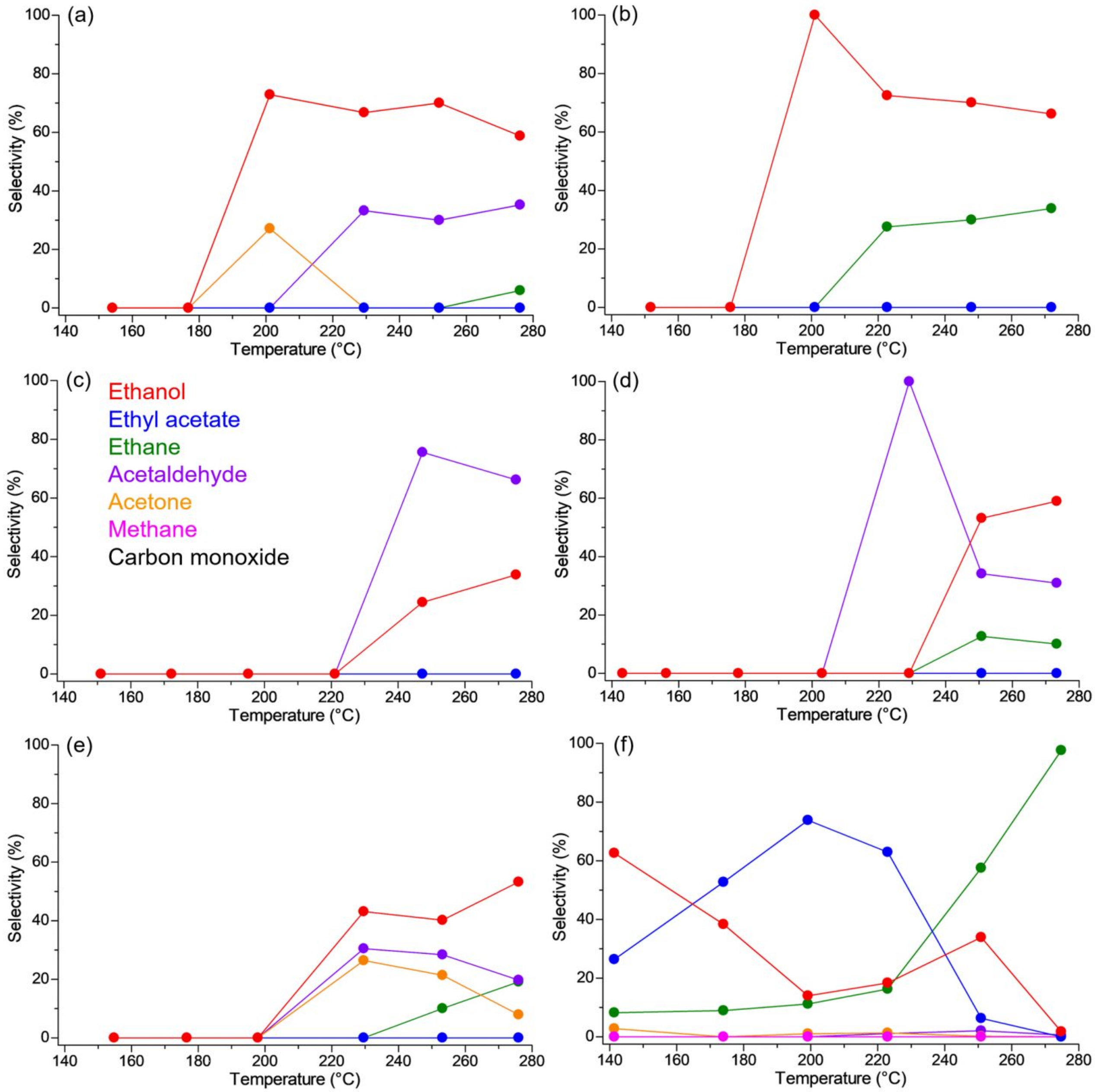

Figure S10. Product selectivity on (a) **Pt/HKUST-1**, (b) **Pt/MIL-101**, (c) **Pt/Zn-MOF-74**, (d) **Pt/Mg-MOF-74**, (e) **Pt/MIL-121**, and (f) Pt/TiO_2.

Table S4. Reaction steps for AH.[2]

(a) Hydrogen dissociation on Pt

$H_{2(g)} + 2^* \rightleftarrows 2H^*$ (*: active site on Pt)

$H^* \rightleftarrows H_{sp}$ (H_{sp}: spillover hydrogen)

(b) Adsorption of AcOH on support

$CH_3COOH_{(g)} + S \rightleftarrows CH_3COOH–S$ (S: support)

(c) Acetaldehyde production by hydrogenation of AcOH

$CH_3COOH–S + H_{sp} \longrightarrow CH_3COOH_2–S$

$CH_3COOH_2–S + H_{sp} \longrightarrow CH_3CHO–S + H_2O_{(g)}$

$CH_3CHO–S \rightleftarrows CH_3CHO_{(g)} + S$

(d) EtOH production by hydrogenation of acetaldehyde

$CH_3CHO–S + H_{sp} \longrightarrow CH_3CHOH–S$

$CH_3CHOH–S + H_{sp} \longrightarrow CH_3CH_2OH–S$

$CH_3CH_2OH–S \rightleftarrows CH_3CH_2OH_{(g)} + S$

(e) Ethane production by hydrogenation of EtOH

$CH_3CH_2OH–S + H_{sp} \longrightarrow CH_3CH_2OH_2–S$

$CH_3CH_2OH_2–S + H_{sp} \longrightarrow CH_3CH_3–S + H_2O_{(g)}$

$CH_3CH_3–S \rightleftarrows CH_3CH_{3(g)} + S$

(f) Possible side reactions

$CH_3COOH + CH_3CH_2OH \longrightarrow CH_3COOCH_2CH_3 + H_2O$ (esterification)

$2CH_3COOH \longrightarrow CH_3COCH_3 + CO_2 + H_2O$ (ketonisation)

$CH_3COOH + H_2 \longrightarrow CH_4 + CO + H_2O$ (decarbonylation)

$CH_3COOH \longrightarrow CH_4 + CO_2$ (decarbonylation)

Table S5. Adsorbed H_2 on **Pt/MOFs** and Pt/TiO_2 determined using H_2-pulse chemisorption after the pretreatment with H_2 at 200 °C.

Pt/MOF	Adsorbed H_2 (μmol g^{-1})
Pt/MIL-125-NH_2	1.89
Pt/UiO-66-NH_2	1.46
Pt/HKUST-1	1.58
Pt/MIL-101	4.65
Pt/Zn-MOF-74	0.98
Pt/Mg-MOF-74	1.02
Pt/MIL-121	1.73
Pt/TiO_2	1.20
Pt/Al_2O_3	2.70

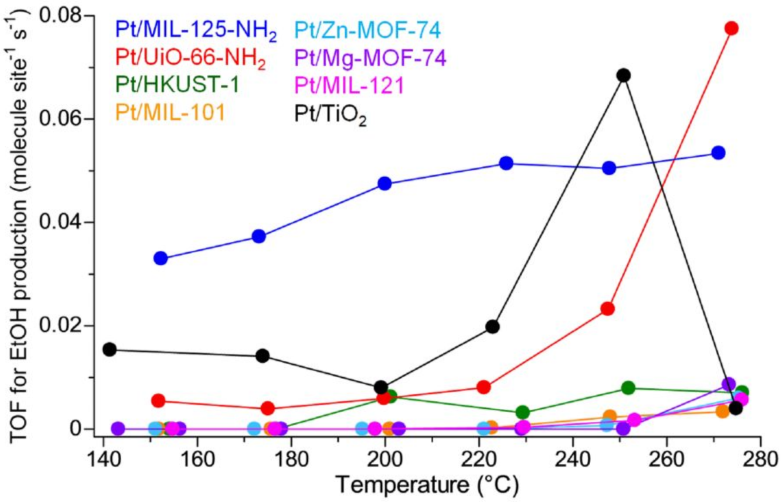

Figure S11. TOF for EtOH production on **Pt/MOFs** and Pt/TiO_2.

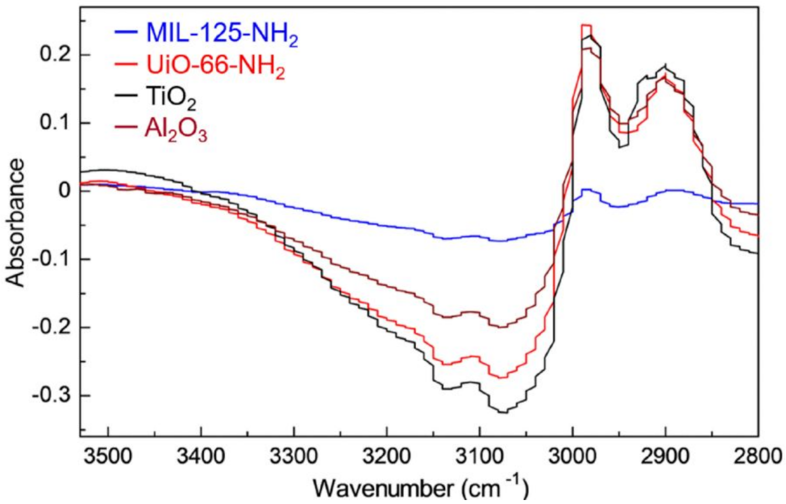

Figure S12. Modulation of IR spectra in **Pt/MIL-125-NH$_2$**, **Pt/UiO-66-NH$_2$**, Pt/TiO_2, and Pt/Al_2O_3, observed at t = 99 s.

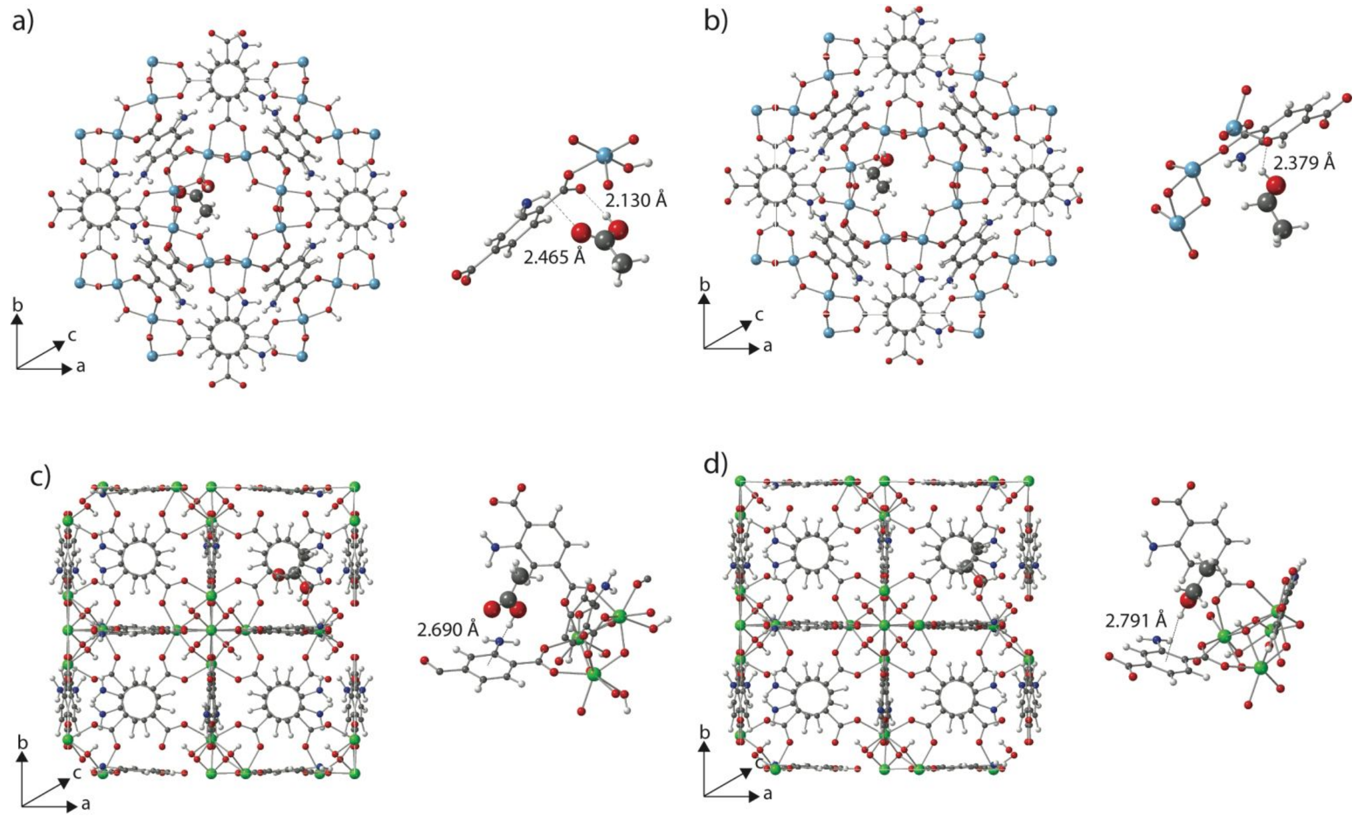

Figure S13. Optimized structures of (a) MIL-125-NH_2 with AcOH, (b) MIL-125-NH_2 with EtOH, (c) UiO-66-NH_2 with AcOH, and (d) UiO-66-NH_2 with EtOH. Magnified views around the adsorbed guest molecules are shown in right. Gray, red, blue, green, cyan, white colors indicate C, O, N, Zr, Ti, and H atoms, respectively.

(Reference)

(1) Yoshimaru, S.; Sadakiyo, M.; Staykov, A.; Kato, K.; Yamauchi, M. *Chem. Commun.* **2017**, *53*, 6720–6723.

(2) Rachmady, W.; Vannice, M. A. *J. Catal.* **2000**, *192*, 322–334.
